# Estimating the Number of Paediatric Fevers Associated with Malaria Infection Presenting to Africa's Public Health Sector in 2007

**DOI:** 10.1371/journal.pmed.1000301

**Published:** 2010-07-06

**Authors:** Peter W. Gething, Viola C. Kirui, Victor A. Alegana, Emelda A. Okiro, Abdisalan M. Noor, Robert W. Snow

**Affiliations:** 1 Spatial Ecology and Epidemiology Group, Department of Zoology, University of Oxford, Oxford, United Kingdom; 2 Malaria Public Health and Epidemiology Group, Centre for Geographic Medicine, KEMRI–University of Oxford–Wellcome Trust Collaborative Programme, Nairobi, Kenya; 3 Centre for Tropical Medicine, Nuffield Department of Clinical Medicine, University of Oxford, Oxford, United Kingdom; London School of Hygiene and Tropical Medicine, United Kingdom

## Abstract

**Background:**

As international efforts to increase the coverage of artemisinin-based combination therapy in public health sectors gather pace, concerns have been raised regarding their continued indiscriminate presumptive use for treating all childhood fevers. The availability of rapid-diagnostic tests to support practical and reliable parasitological diagnosis provides an opportunity to improve the rational treatment of febrile children across Africa. However, the cost effectiveness of diagnosis-based treatment polices will depend on the presumed numbers of fevers harbouring infection. Here we compute the number of fevers likely to present to public health facilities in Africa and the estimated number of these fevers likely to be infected with *Plasmodium falciparum* malaria parasites.

**Methods and Findings:**

We assembled first administrative-unit level data on paediatric fever prevalence, treatment-seeking rates, and child populations. These data were combined in a geographical information system model that also incorporated an adjustment procedure for urban versus rural areas to produce spatially distributed estimates of fever burden amongst African children and the subset likely to present to public sector clinics. A second data assembly was used to estimate plausible ranges for the proportion of paediatric fevers seen at clinics positive for *P. falciparum* in different endemicity settings. We estimated that, of the 656 million fevers in African 0–4 y olds in 2007, 182 million (28%) were likely to have sought treatment in a public sector clinic of which 78 million (43%) were likely to have been infected with *P. falciparum* (range 60–103 million).

**Conclusions:**

Spatial estimates of childhood fevers and care-seeking rates can be combined with a relational risk model of infection prevalence in the community to estimate the degree of parasitemia in those fevers reaching public health facilities. This quantification provides an important baseline comparison of malarial and nonmalarial fevers in different endemicity settings that can contribute to ongoing scientific and policy debates about optimum clinical and financial strategies for the introduction of new diagnostics. These models are made publicly available with the publication of this paper.

*Please see later in the article for the Editors' Summary*

## Abstract 2

Peter Gething and colleagues compute the number of fevers likely to present to public health facilities in Africa and the estimated number of these fevers likely to be infected with *Plasmodium falciparum* malaria parasites.

## Editors' Summary

**Background:**

Malaria —an infectious parasitic disease transmitted to people through the bite of an infected mosquito —kills about one million people (mainly children living in sub-Saharan Africa) every year. Although several parasites cause malaria, *Plasmodium falciparum* is responsible for most of these deaths. For the past 50 years, the main treatments for *P. falciparum* malaria have been chloroquine and sulfadoxine/pyrimethamine. Unfortunately, parasitic resistance to these “monotherapies” is now widespread and there has been a global upsurge in the illness and deaths caused by *P. falciparum*. To combat this increase, the World Health Organization recommends artemisinin combination therapy (ACT) for *P. falciparum* malaria in all regions with drug-resistant malaria. In ACT, artemisinin derivatives (new, fast-acting antimalarial drugs) are used in combination with another antimalarial to reduce the chances of *P. falciparum* becoming resistant to either drug.

**Why Was This Study Done?:**

All African countries at risk of *P. falciparum* have now adopted ACT as first-line therapy for malaria in their public clinics. However, experts are concerned that ACT is often given to children who don't actually have malaria because, in many parts of Africa, health care workers assume that all childhood fevers are malaria. This practice, which became established when diagnostic facilities for malaria were very limited, increases the chances of *P. falciparum* becoming resistant to ACT, wastes limited drug stocks, and means that many ill children are treated inappropriately. Recently, however, rapid diagnostic tests for malaria have been developed and there have been calls to expand their use to improve the rational treatment of African children with fever. Before such an expansion is initiated, it is important to know how many African children develop fever each year, how many of these ill children attend public clinics, and what proportion of them is likely to have malaria. Unfortunately, this type of information is incompletely or unreliably collected in many parts of Africa. In this study, therefore, the researchers use a mathematical model to estimate the number of childhood fevers associated with malaria infection that presented to Africa's public clinics in 2007 from survey data.

**What Did the Researchers Do and Find?:**

The researchers used survey data on the prevalence (the proportion of a population with a specific disease) of childhood fever and on treatment-seeking behavior and data on child populations to map the distribution of fever among African children and the likelihood of these children attending public clinics for treatment. They then used a recent map of the distribution of *P. falciparum* infection risk to estimate what proportion of children with fever who attended clinics were likely to have had malaria in different parts of Africa. In 2007, the researchers estimate, 656 million cases of fever occurred in 0–4-year-old African children, 182 million were likely to have sought treatment in a public clinic, and 78 million (just under half of the cases that attended a clinic with fever) were likely to have been infected with *P. falciparum*. Importantly, there were marked geographical differences in the likelihood of children with fever presenting at public clinics being infected with *P. falciparum*. So, for example, whereas nearly 60% of the children attending public clinics with fever in Burkino Faso were likely to have had malaria, only 15% of similar children in Kenya were likely to have had this disease.

**What Do These Findings Mean?:**

As with all mathematical models, the accuracy of these findings depends on the assumptions included in the model and on the data fed into it. Nevertheless, these findings provide a map of the prevalence of malarial and nonmalarial childhood fevers across sub-Saharan Africa and an indication of how many of the children with fever reaching public clinics are likely to have malaria and would therefore benefit from ACT. The finding that in some countries more than 80% of children attending public clinics with fever probably don't have malaria highlights the potential benefits of introducing rapid diagnostic testing for malaria. Furthermore, these findings can now be used to quantify the resources needed for and the potential clinical benefits of different policies for the introduction of rapid diagnostic testing for malaria across Africa.

**Additional Information:**

Please access these Web sites via the online version of this summary at http://dx.doi.org/10.1371/journal.pmed.1000301.

- Information is available from the World Health Organization on malaria (in several languages) and on rapid diagnostic tests for malaria
- The US Centers for Disease Control and Prevention provide information on malaria (in English and Spanish)
- MedlinePlus provides links to additional information on malaria (in English and Spanish)
- Information on the global mapping of malaria is available at the Malaria Atlas Project
- Information is available from the Roll Back Malaria Partnership on the global control of malaria (in English and French) and on artemisinin combination therapy

## Introduction

Between 2003 and 2009 all African countries at risk of *P. falciparum* switched to artemisinin-based combination therapy (ACT) as first-line therapy for uncomplicated malaria in their public health sectors [1]. Access to these essential drugs remains poor, however, with the World Health Organization reporting that only one-sixth of fever cases in children under 5 y of age was treated with an ACT in 2008 (in ref. [2], see Table 3.8). Paradoxically, the prevailing doctrine to treat all fevers as malaria, promoted during the era of widely available mono-therapies and limited diagnostic facilities, has lead to the systematic overdiagnosis of malaria in many parts of Africa [3]–[6]. This status quo leads to the inappropriate distribution and prescription of limited ACT stocks and jeopardizes the appropriate case management of nonmalarial fevers [7]–[9]. The single largest policy change likely to impact upon the quality of case management for fevers, and on future ACT requirements, is the implementation of improved parasitological diagnosis to support appropriate prescription practices in all age groups [10],[11]. Some argue that the expanded use of rapid diagnostic tests (RDTs) by prescribers at the point of care will significantly change diagnosis and treatment of febrile patients [10],[12]–[15], increase the geographic reach of parasite diagnostic services, and replace the lack of prescriber confidence in poor routine microscopy [3],[16]–[21]. Whilst there is broad agreement that a long-term goal should be the laboratory or RDT-based confirmation of all suspected malaria cases prior to treatment with an antimalarial, considerable debate continues surrounding the implications of such a transition under current conditions [10],[22]–[24].

Identifying optimum strategies for the phased introduction of new diagnostic policies requires reliable information on a range of spatially distributed public health metrics. Of particular importance is an understanding of the volume of fever cases currently presenting to health systems and the extent to which these fevers are associated with *P. falciparum* parasitemia. Routine approaches to estimating such metrics in government-supported clinics rely mainly on data from imperfect health management information systems [25]–[27]. Such data are fundamentally limited in many African settings and characterized by sporadic and often inaccurate reporting [27],[28]. An alternative approach is to spatially triangulate more robust facility- and community-based survey data to establish plausible rates of fever in the population, the proportion expected to attend clinics, and the proportion likely to have malaria, thus providing estimates that are entirely independent of imperfect health system data [29],[30]. In an earlier morbidity modelling exercise that preceded the universal change in ACT policy, data on reported fevers from national sample surveys were used to estimate treatment burden for fevers across Africa [30]. Data were reconciled at national levels for population estimates for the year 2000 against crude simulations of stable malaria risk on the basis of theoretical climate suitability for transmission. It is now possible to assemble more contemporary data on fever prevalence, reconciled at higher spatial resolutions and matched to better models of malaria transmission risk. Here we use this approach to estimate and compare two fundamental metrics: the total number of paediatric fever cases seeking treatment in the public sector, and the number of those cases likely to have malaria.

## Methods

### Model Overview

**Figure 1:**
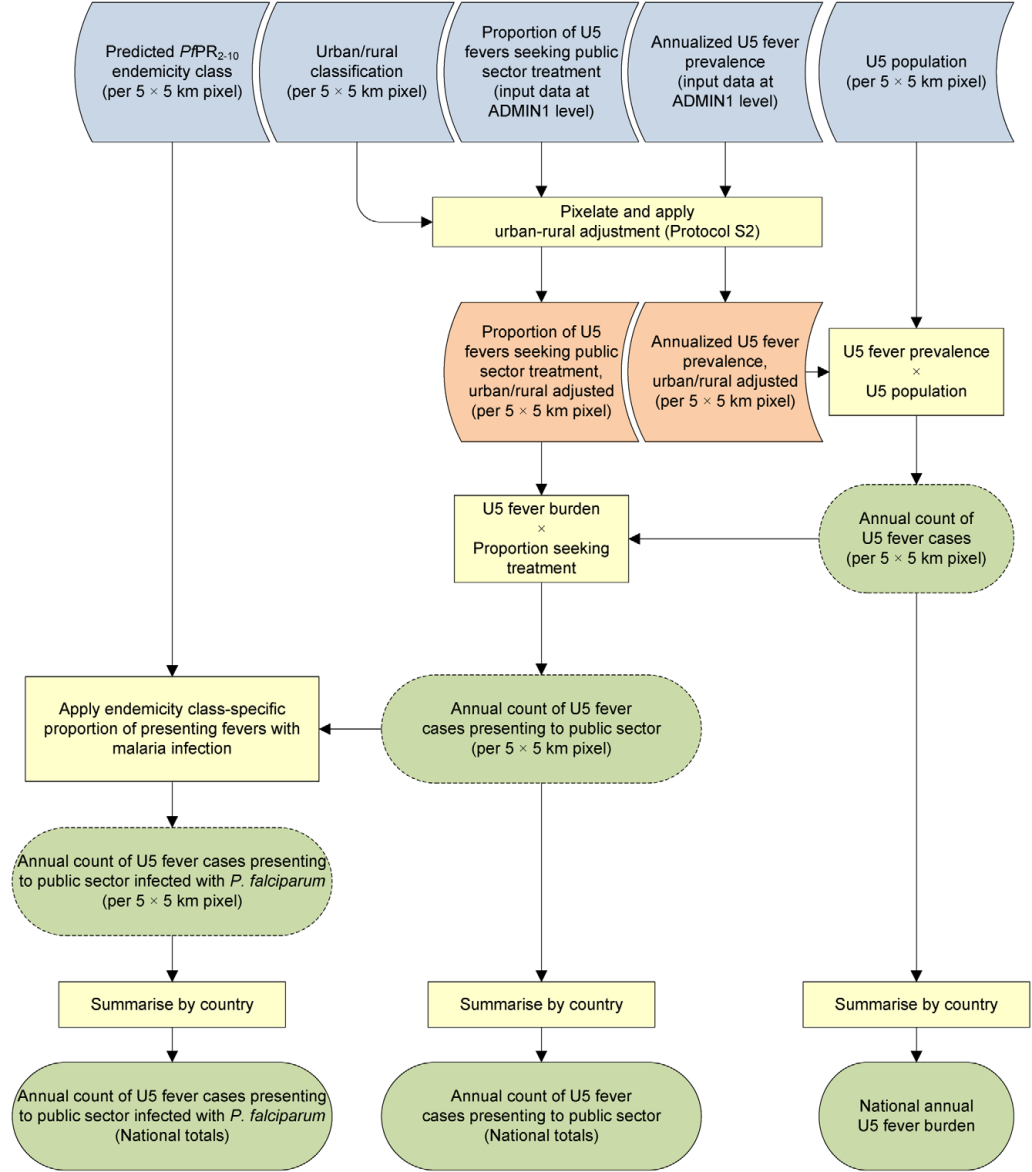
Schematic overview of mapping procedures and methods. Blue rods describe input data; yellow boxes denote operations in a geographical information system; orange rods denote adjusted data; green rods indicate output data, with dashed lines denoting intermediate output and solid lines final outputs. U5, children aged under 5 y old.

We used a simple pathway framework to derive the annualized population of febrile children in Africa presenting to public health facilities, and the proportion likely to have malaria: (1) we defined the 14-d period prevalence of reported fevers among children aged 0–4 y by subnational administrative units, applying an urban–rural adjustment factor and transforming to an annualized burden using population projections for the year 2007; (2) we defined for each administrative unit the proportion of fevers that seek care from a public health facility, adjusting for urban–rural differences; (3) the annualized fever burdens presenting to public health facilities were structured according to four malaria transmission risk strata; and finally, (4) we estimated the proportion of febrile children presenting to clinics that had malaria infections according to the different malaria transmission classifications. These steps are discussed in detail below and summarized schematically in Figure 1.

### Assembling Data on Fever Prevalence and Treatment Seeking among Children Aged 0–4 Years

We identified report and raw data from various national sample surveys where the history of fever in the preceding 14 d was reported from interviews with mothers and caretakers of resident children aged 0–4 y. These data form part of monitoring and evaluation surveys undertaken approximately every 3–5 y across Africa as part of national Demographic and Health Surveys (DHS; http://www.measuredhs.com/), Multiple Indicator Cluster Surveys (MICS; http://www.unicef.org/statistics/index_24302.html), or more recently national Malaria Indicator Surveys (MIS; http://www.rollbackmalaria.org/mechanisms/merg), and are presented in full in Table S1. The Monitoring and Evaluation Reference Group of the Roll Back Malaria (RBM) initiative has promoted the use of these data to serially track progress against several key RBM targets including insecticide-treated net coverage, coverage of intermittent presumptive treatment in pregnancy, and access to effective antimalarial treatment within 48 h of the onset of symptoms in young children [31]. The denominators used in the RBM treatment indicators are all children reported to have had a fever in the last 14 d. The multistage sampling design from first-level administrative levels (e.g. province, state, or governorate) to national census defined “enumeration clusters” is common to all these surveys, and sample sizes are calculated on an ability to provide precision in health and population indicators at the first-level administrative unit (hereafter ADMIN1). We have reconstructed information reported at ADMIN1 levels on the numbers of children aged less than 5 y investigated, the number reported as having had a fever in the 14 d preceding the survey, and the number using a public health facility to treat the fever. 2-wk period fever prevalence and treatment-seeking rates were derived directly from these data. Period prevalence values were then annualized by multiplying by 26. This simple approach assumed that most surveys were undertaken at a time representative of fever incidence across an average 12 mo, and also that most fevers began and ended during the 2-wk survey period. These annualized prevalence data were rasterised using ArcView 3.2 (ESRI Inc.) into a 5×5-km resolution surface in which each pixel contained the values reported at ADMIN1 level.

We have selected the most recent national sample survey for each country that allowed the most complete definition of fever period prevalence and the proportion of fevers seeking treatment from public sector services (Table S1). For four countries no information was available on fever prevalence (Eritrea, Botswana, South Africa, and Cape Verde). The surveys undertaken in the Comoros (2000), Equatorial Guinea (2000), São Tomé and Principe (2000), Sierra Leone (2005), Sudan (North and South, 2005), Mauritania (2007), and Togo (2006) did not document the sources of treatment sought for children reported as having a fever in the last 14 d, so the treatment-seeking data for reported acute respiratory tract infections were used as a surrogate. The surveys in Malawi in 2006 and Senegal in 2008–2009 did not report treatment sources for fevers so we have assumed a similar pattern to the fever treatment-seeking patterns described in the DHS survey in 2000 for Malawi and the 2006 MIS for Senegal. In some countries more recent data on fever prevalence have been collected as part of national sample surveys but information is not currently available in the public domain (Namibia, MIS 2009; Mozambique, MIS 2007; Liberia, MIS 2007; Ethiopia, MIS 2007; Rwanda, DHS 2007–2008) and earlier surveys have been used. Nevertheless the majority of the data were assembled after 2004 with the exception of Central African Republic (1994–1995), Comoros (2000), Equatorial Guinea (2000), São Tomé and Principe (2000), Gabon (2000–2001), Nigeria (2003), Mozambique (2003), Madagascar (2003–2004), and Chad (2004). For these countries more recent data were not available in the public domain and we have assumed that fever prevalence and sources of treatment for fevers had not changed significantly over time.

### Defining 0–4 Year Population Distributions and Urban–Rural Boundaries

The Global Rural Urban Mapping Project (GRUMP) alpha version provides gridded population counts and population density estimates for the year 2000, both adjusted and unadjusted to the United Nations' national population estimates [32]. The adjusted population counts were projected forward to create seven further population count surfaces for each year from 2001–2007 by applying national, medium variant, intercensal growth rates by country [33], using methods described previously [34]. These population counts were then stratified nationally by age group using United Nations' defined population age structures [33] to obtain 0–4 y of age population count surfaces at 5×5-km resolution for the year 2007. We also obtained the GRUMP urban extents product (GRUMP-UE), which identifies urban areas across a corresponding 5×5-km grid on the basis of principally night-time lights satellite imagery supplemented with data derived from tactical pilotage charts and known settlement points [35]–[37].

### Adjusting Fever Prevalence and Treatment-Seeking Rates for Urban–Rural Differences

Both the prevalence of fevers in children and the fraction of fever cases that seek care in the public health sector are potentially different in urban versus rural areas. In addition to period prevalence and treatment-seeking data reported at ADMIN1, many of the assembled national surveys reported national level summaries of both variables stratified by urban and rural areas, allowing a national level urban–rural ratio to be derived for each. For these countries we used the GRUMP-UE surface to identify urban and rural areas. Using the national level urban–rural ratios we adjusted the reported prevalence and treatment-seeking rates on a pixel-by-pixel basis within each ADMIN1 unit in such a way that the population-weighted mean for each unit was preserved, but the reported value in each pixel was adjusted upward or downward according to its urban–rural status and the national urban–rural ratio. This procedure is explained in more detail in Protocol S1.

### Estimating Spatially Varying Malaria Risk Classes

**Figure 2:**
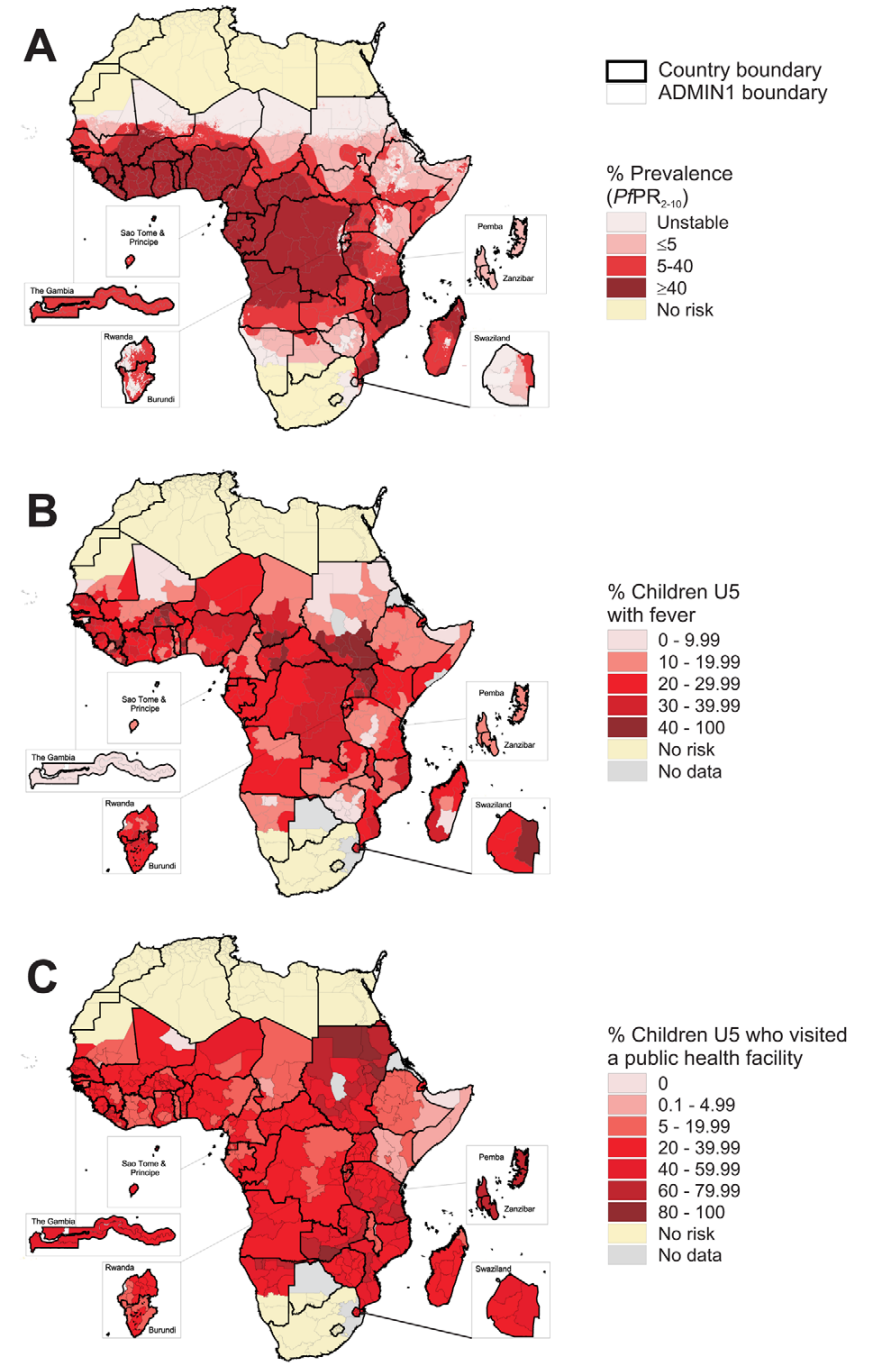
Transmission intensity, fevers, and care seeking for fever across Africa. (**A**) Predicted transmission intensity across Africa. Transmission is classified into areas of risk free, unstable, and stable transmission on the basis of country-reported case data and the limiting effects on transmission of aridity and low temperatures [38]. The latter class is further divided into low, medium, and high transmission settings from a model-based geostatistical prediction of *P. falciparum* prevalence in the epidemiologically informative 2-y up to 10-y age range, *PfPR_2–10_* [34]. (**B**) 14-d period prevalence of reported fevers among children aged 0–4 y derived from national sample survey data (yellow, no risk; grey, no data). (**C**) Proportion of paediatric fevers using a public health facility at some stage of the illness to treat the fever (yellow, no risk; grey, no data). Footnote: The reference ADMIN1 digital boundaries for Africa were obtained through a combination of data from the United Nations Geographic Information Working Group, Second Administrative Level Boundary project (UNGIWG-SALB [56]) and the Food & Agriculture Organization - Global Administrative Units Layers (FAO-GAUL [57]). These boundary units matched reported information on fever prevalence for 31 of 42 national survey reports assembled. For Angola, Burundi, Central African Republic, Chad, Congo, Gabon, Guinea Bissau, Mauritania, and Nigeria nonstandard ADMIN1 units were reported by the national sample surveys and these were digitized using ArcGIS 9.3 (ESRI, Inc.) to replace existing ADMIN1 boundaries and thus create a single fever spatial reporting surface, similar to recent approaches to assemble mosquito net use from national survey data [58].

We have recently published revised global limits of unstable and stable *P. falciparum* infection risk [38] and a modelled mapped distribution of the intensity of *P. falciparum* within the stable margins of transmission based upon infection prevalence among children aged 2 up to 10 y (*Pf*PR_2–10_) [34]. In brief, data on national case reporting, national and international medical intelligence, climate, and aridity were used to define conservatively the margins of stable and unstable *P. falciparum* transmission [38]. Stable malaria transmission was assumed to represent a minimum average of one clinical case per 10,000 population per annum (pa) in a given administrative unit; unstable malaria transmission was used to define areas where transmission was biologically plausible and/or had been documented but where incidence was likely to be less than one case per 10,000 population pa, in Africa largely areas where aridity limits the survival of larvae and causes desiccation of adult vectors; finally no transmission was assumed where assembled intelligence stated no malaria risk because (1) national reporting systems had, over several years, not reported a single *P. falciparum* clinical case, or (2) where temperature was too low for sporogony to complete within the average lifespan of the local dominant vector species. Within the stable transmission margins, empirical community survey data on parasite prevalence were assembled and geolocated to provide the basis for an urban–rural and sample-size adjusted geospatial model within a Bayesian framework to interpolate a continuous space-time posterior prediction of *Pf*PR_2–10_ for every 5×5-km pixel for the year 2007 [34]. This model also generated classified output that assigned each pixel to one of four malaria endemicity classes: malaria free or unstable, *Pf*PR_2–10_ ≤5%; *Pf*PR_2–10_ >5% to <40%; and *Pf*PR_2–10_ ≥40% (Figure 2A). These classifications of stable transmission correspond to ranges of *Pf*PR that have been proposed to select suites of interventions at scale to reach elimination targets at different time periods [39],[40] and have a congruence with fever infection prevalence thresholds used in cost-effectiveness models to select RDTs [7],[9],[41].

### Computing the Annualized Health System Burden of Fevers Stratified by Endemicity Class

We derived an estimate of the annual number of paediatric fever cases occurring in each 5×5-km pixel by multiplying its urban–rural adjusted annualized fever prevalence with its projected 2007 0–4-y-old population. Each pixel value was then multiplied by the corresponding urban–rural adjusted estimate for the proportion of fevers accessing a public health facility, thus converting our total child fever burden estimate for each pixel into the burden likely to present to the public health service. We then combined these pixel-level estimates of fevers presenting to health systems with the mapped 5×5-km *Pf*PR_2–10_ grid surface, along with digital national boundaries, allowing the summation of fever burden estimates stratified by country and by malaria endemicity class.

### Defining the Proportion of Fevers Presenting to Clinics with Confirmed Malaria

We performed a PubMed search using the Medical Subject Headings (MeSH) terms “malaria diagnosis Africa” on titles, abstracts, and full text and selected all publications since 2000 (Protocol S2). The search resulted in 67 temporally and/or spatially independent reports of varying definitions of presumed malaria and the proportion of these that were confirmed through detailed microscopy as having evidence of *P. falciparum* malaria. The data covered 20 countries and were represented by 13 drug trials where information was provided on the numbers of patients screened and those found positive for malaria infection, 12 reports of surveillance where expert slide reading was available, 14 audits of clinical practice where patients were rechecked for parasitology on leaving the clinic, 22 studies of RDT or immunochromatographic test (ICT) versus expert microscopy performance, six other study sites where diagnostic algorithms were tested, and one study of patient drug adherence. Study sites were located using a variety of digital place-name gazetteers and mapping resources including the Encarta Encyclopedia (Microsoft, 2007), Google Earth (Google, 2009), and other sources described previously [42]. The site's longitude and latitude were used to match the 5×5-km grid square of the study to the predicted posterior mean *Pf*PR_2–10_ for 2007 [34]. Matching was done spatially but not temporally, i.e. to 2007 rather than the year of the study. The spatially matched estimate of *PfPR_2–10_* was used to classify each survey site into one of the four endemicity classes described earlier to explore the relationship between underlying endemicity and the likelihood that paediatric fevers attending clinics were positive for *P. falciparum*. All data, their inclusion criteria, and age ranges are shown in Protocol S2.

## Results

### Period Prevalence of Fever

Between 103 and 10,080 interviews with mothers or caretakers were undertaken in each of 336 ADMIN1 units to investigate fever period prevalence among children aged 0–4 y providing information on 281,426 children. A total of 70,666 fevers were identified across the 42 countries and 14-d period prevalence of fever ranged from 0.7% in Kidal, Mali to 53.4% in West Equatoria in Southern Sudan (Figure 2B). Fever prevalence was less than 5% in only eight ADMIN1 units, including three in Zimbabwe, two in Northern Sudan, and one each in Mali, The Gambia, and São Tomé and Principe. The median fever period prevalence across the entire ADMIN1 range was 23.8% (interquartile range [IQR] 15.2%–30.6%). All assembled data on fever prevalence are presented nationally in Table S1. Overall there was no discernible relationship between the intensity of transmission and the proportion of children reported as having a fever in the last 2 wk (unpublished results). Nationally reported ratios for urban versus rural fever prevalence are presented in Protocol S1 and ranged from 0.55 in Uganda to 1.29 in São Tomé and Principe, with a median ratio of 0.87.

Transforming the period prevalence of fever to an annualized estimate among the resident projected populations of children, we estimate that there were 655.6 million reported fevers in 2007 among the 42 countries included in this analysis (i.e., excluding Eritrea, South Africa, Botswana, and Cape Verde). Eleven ADMIN1 units were estimated to account for more than 10 million fevers each: six in Nigeria, three in Ethiopia, and two in Democratic Republic of Congo (DRC). These 11 areas of Africa alone contributed 32% of the entire estimated African paediatric fever burden in 2007.

### Treatment Seeking for Childhood Fevers

Two ADMIN1 units reported none of the febrile children accessing care in the formal public health sector: Kidal in Mali, where only two fevers were reported, and none among the 112 fevers reported in North West Somalia, consistent with the prolific private medicine sector in this area [43] and the fact that the public sector in Somalia has largely been destroyed during the civil conflict. Other countries with a low (<10%) public health facility use for fevers included Côte D'Ivoire, Ethiopia, and Chad. The highest (≥50%) use of public health services for fever treatment was reported for Mozambique, Northern Sudan, Liberia, Djibouti, Southern Sudan, Namibia, Tanzania, and Zambia. Use of public services exceeding 75% was reported in some areas of Sudan, Tanzania, and Zambia. All assembled data on treatment seeking for fever are presented nationally in Table S1. The median public health service use for fever treatment was 32.5% (IQR 22.7%–45.2%) and the diversity across the continent is shown in Figure 2C. Nationally reported urban–rural ratios in the use of public health services are presented in Protocol S1 and ranged from 0.45 in Burkina Faso to 5.05 in Chad, with a median ratio of 1.19.

### Proportion of Paediatric Fevers at Clinics Associated with *P. falciparum* Infection

**Figure 3:**
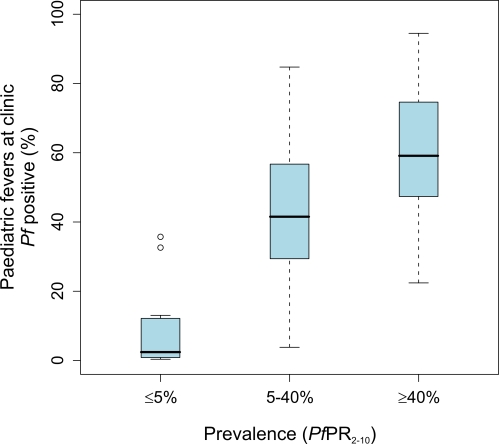
Risks of febrile children being infected when presenting to clinics within three epidemiological strata of unstable/≤5% *PfPR_2–10_*, >5% to <40% *PfPR_2–10_*, and ≥40% *PfPR_2–10_*. The box indicates the IQR (25% and 75%); the thick line within the box represents the median; the whiskers represent the 2.5% and 97.5% centiles; and outliers are plotted as circles outside this range.

The 67 independent estimates of the proportion of febrile children attending clinics found to be positive with expert microscopy covered a wide range of transmission conditions predicted by the 5×5-km modelled map surface (sites spanned *PfPR_2–10_* 0%–68%). No sites were available in areas classified as risk-free, and we assumed that no fevers at clinics in these areas would be infected with *P. falciparum*. Three study sites were located in the unstable malaria transmission category, 11 sites in areas where stable transmission is ≤5% *PfPR_2–10_*, 32 sites in areas where transmission ranged from >5% to <40% *PfPR_2–10_*, and 21 sites in the highest stable transmission category (≥40% *PfPR_2–10_*). The median proportions of fevers infected were similar between the endemicity classes of unstable and ≤5% *PfPR_2–10_* (see Protocol S2), and these 14 study sites have been combined in Figure 3 to examine broad differences in fever infection between locations of differing endemicity. Under the lowest transmission intensity classification the median proportion of infected fevers was 3.3% (IQR 0.5%–12%). Among children presenting to clinics in the medium transmission intensity class the median proportion harbouring infection was 41.5% (IQR 29%–57%). Among fevers presenting to clinics located in the highest transmission intensity areas the median infection rate was 59.0% (IQR 47%–75%). Despite overlapping ranges of fever infection risks among the medium and high transmission classes, the endemicity classification provides a legitimacy to the intuitive prior expectation that areas of higher parasite transmission are likely to see more children at clinics with peripheral infections. It is important to note, however, that in a given proportion of parasitemic fever cases, the fever will not be caused by the malaria infection. We make no attempt here to attribute causation or to quantify this malaria attributable fraction, but note that this proportion will tend to decrease systematically with rising endemicity. Importantly, however, for diagnostic-based treatment policies all infected fevers are likely to be treated as malaria.

### Burden of Fevers Associated with Malaria in Public Health Facilities

Applying the urban–rural adjusted ADMIN1-specific treatment-seeking estimates to the projected annualized burdens, we estimate that of the 655.5 million fever events affecting children in Africa in 2007, approximately 182.4 million will have presented at public health facilities, of which 78.3 million were likely to have been positive for *P. falciparum*. As part of a sensitivity analysis (described in full in Protocol S3), the number of infected fevers presenting at public health facilities was recalculated under more extreme scenarios where the observed 0.25 and 0.75 quantile infection rate was used for each endemicity class rather than the median value. This resulted in a plausible range of 59.8–103.4 million fevers at clinics likely to be positive for *P. falciparum*, reflecting our uncertainty in the infection rate in different settings. Correspondingly, we estimate that 104.1 (79.0–122.6) million paediatric fevers attending public clinics in 2007 were not accompanied by a malaria infection.

### Malarial and Nonmalarial Fevers by Country and Endemicity Setting

Health system decisions on malaria diagnostic and case management practices are likely to be made primarily at the national level and would ideally be informed by within-country variations in endemicity. Table 1 presents summary estimates for each country of the total number of paediatric fevers attending public health facilities in 2007, and the number of those fevers predicted to be associated with *P. falciparum* infection. Across Africa, we estimate in areas of zero, unstable/low (*PfPR_2–10_*≤5%), moderate (*PfPR_2–10_*>5% to <40%), and high (*PfPR_2–10_*≥40%) risk, that 13.5 million, 22.4 million, 50.7 million, and 95.8 million paediatric fevers, respectively, will have sought care from a public health facility of which zero, 0.7 million (range 0.1–2.7 million), 21.0 million (range 14.7–28.9 million), and 56.5 million (range 45.0–71.8 million), respectively, would have been accompanied by *P. falciparum* infection.

**Table 1:** Estimated total and *P. falciparum* positive paediatric fevers attending public health facilities in Africa in 2007.

Country^a^	All U5 Fevers Attending PHFs	U5 Fevers Attending PHFs with *P. falciparum* Infection		
		All Areas (% of Attending Fevers)	Where *Pfpr_2–10_* <40% (% of *P. falciparum* Positives)	Where *Pfpr_2–10_*≥40% (% of *P. falciparum* Positives)
**Angola**	6,176	3,299 (53.4)	286 (8.7)	3,013 (91.3)
**Benin**	1,934	1,116 (57.7)	52 (4.7)	1,064 (95.3)
**Burkina Faso**	1,499	884 (59.0)	1 (0.1)	883 (99.9)
**Burundi**	1,407	401 (28.5)	301 (75.2)	99 (24.8)
**Cameroon**	1,971	1,061 (53.8)	157 (14.8)	905 (85.2)
**CAR**	933	541 (58.0)	21 (3.8)	521 (96.2)
**Chad**	1,827	660 (36.1)	631 (95.6)	29 (4.4)
**Comoros**	39	1 (3.1)	1 (100.0)	0 (0)
**Congo**	690	407 (59.0)	0 (0)	407 (100.0)
**Côte D**'**Ivoire**	2,132	1,247 (58.5)	0 (0)	1,247 (100.0)
**Djibouti**	131	4 (3.0)	4 (100.0)	0 (0)
**DRC**	20,969	11,335 (54.1)	471 (4.2)	10,864 (95.8)
**Eq. Guinea**	95	54 (56.3)	4 (8.2)	49 (91.8)
**Ethiopia**	6,939	578 (8.3)	578 (100.0)	0 (0)
**Gabon**	208	117 (55.9)	14 (12.4)	102 (87.6)
**Gambia**	154	63 (40.9)	63 (100.0)	0 (0)
**Ghana**	3,847	2,169 (56.4)	110 (5.1)	2,058 (94.9)
**Guinea**	3,123	1,645 (52.7)	460 (27.9)	1,186 (72.1)
**Guinea Bissau**	113	46 (40.4)	46 (100.0)	0 (0)
**Kenya**	11,821	1,724 (14.6)	1,502 (87.1)	222 (12.9)
**Liberia**	1,257	735 (58.5)	0 (0)	735 (100.0)
**Madagascar**	3,629	1,623 (44.7)	775 (47.8)	847 (52.2)
**Malawi**	1,668	857 (51.4)	295 (34.4)	563 (65.6)
**Mali**	2,507	1,334 (53.2)	119 (8.9)	1,214 (91.1)
**Mauritania**	85	12 (14.1)	11 (92.2)	1 (7.8)
**Mozambique**	10,360	5,202 (50.2)	1,056 (20.3)	4,145 (79.7)
**Namibia**	423	51 (12.0)	51 (100.0)	0 (0)
**Niger**	4,480	1,923 (42.9)	1,321 (68.7)	602 (31.3)
**Nigeria**	35,781	20,345 (56.9)	1,540 (7.6)	18,805 (92.4)
**Rwanda**	2,170	593 (27.3)	579 (97.6)	14 (2.4)
**Senegal**	4,165	1,269 (30.5)	1,269 (100.0)	0 (0)
**Sierra Leone**	2,164	1,233 (57.0)	0 (0)	1,233 (100.0)
**Somalia**	87	21 (23.5)	19 (93.2)	1 (6.8)
**ST & P**	15	6 (40.2)	6 (100.0)	0 (0)
**Sudan**	12,563	1,786 (14.2)	1,742 (97.5)	45 (2.5)
**Swaziland**	371	26 (7.0)	26 (100.0)	0 (0)
**Tanzania**	15,140	5,952 (39.3)	3,496 (58.7)	2,456 (41.3)
**Togo**	525	310 (59.0)	0 (0)	310 (100.0)
**Uganda**	13,002	5,899 (45.4)	3,156 (53.5)	2,743 (46.5)
**Zambia**	5,049	1,746 (34.6)	1,612 (92.4)	133 (7.6)
**Zimbabwe**	985	34 (3.4)	34 (100.0)	0 (0)
**Total**	182,433	78,306 (42.9)	21,810 (27.9)	56,496 (72.1)

The latter estimates are also shown stratified by areas of low or moderate (*PfPR_2–10_* <40%) and high (*PfPR_2–10_* ≥40%) endemicity. All totals are given in '000s.

a Four African countries are excluded because of unavailable data: Eritrea, South Africa, Botswana, and Cape Verde.

Abbreviations: CAR, Central African Republic; DRC, Democratic Republic of Congo; PHF, public health facility; ST & P, São Tomé and Principe; U5, children under 5 y old.

An additional exercise was undertaken to assess the sensitivity of these results to reverting to a simpler analysis that made no adjustments for potential urban–rural differences in fever prevalence and treatment-seeking behaviour (presented in full in Protocol S3). The overall effect of the adjustment was modest, with the mean effect size at national level less than 1%, although this varied considerably between countries as a function of their observed urban–rural differentials and endemicity environments.

## Discussion

We have used an assembly of spatially distributed data on African children, fevers, and treatment-seeking behaviour, along with comparisons of malaria infection prevalence amongst those fevers reaching clinic in different endemicity settings to estimate that 656 million fevers occurred in African 0–4 y olds in 2007. 182 million (28%) of these febrile events were likely to have resulted in a visit to a public sector clinic of which 78 million (43%) were likely to have been infected with *P. falciparum* (range 60–103 million).

As the scale-up of ACT provision to front-line health facilities in sub-Saharan Africa continues, and optimum strategies for improving routine diagnostic practices are sought, the establishment of reliable baseline estimates of key public health metrics becomes increasingly important. We have used national sample survey data on fever period prevalence as the entry point to define children seeking treatment in public health facilities managed by national governments who have the responsibility for providing effective case management via appropriate diagnostic strategies and adequate drug and commodity provision. These survey data have been collected from 42 African countries across time periods spanning the escalation of ACT policy change on the continent. We have modelled spatially the expected subnational fever contact rate with public health services and apportioned these fevers into those with and without a malaria infection according to varied malaria transmission intensity conditions.

The results presented here provide a means to quantify the anticipated drug and commodity requirements under different diagnostic policy scenarios. For example, if a policy of presumptive treatment of all fevers had been universally adhered to in 2007, 182 million ACT treatments would have been required to treat children under 5 y presenting to front-line health facilities across Africa, but 104 million (57%) of these may have been prescribed to children without evidence of infection, highlighting the potential for policies supporting diagnosis to substantially reduce overprescription to febrile children. A utopian scenario in which every prescriber adheres to the results of a diagnostic test is, however, unlikely to be achieved universally. The current and theoretical future adherence to recommended diagnostic policies, the availability of both drugs and diagnostic kits, the attitude of prescribers to test results, the expectations of patients, and temporal changes to underlying endemicity and treatment-seeking behaviours must all be considered if the full implications of different policy scenarios are to be evaluated. Nevertheless, the starting point for any such evaluation is a set of baseline metrics that quantify the magnitude of the current treatment burden for fever in Africa and the spatially varying contribution of malaria to this burden.

A particularly important inference from the results presented in this study is that any new policy on diagnostics is likely to be most effective if geographic heterogeneity in infection risk and parasitemia in fevers is considered. As expected, we found the largest differences between malarial and nonmalarial fever burdens in countries where the dominant transmission intensity is unstable or low and fever incidence and use of public services are high. The proportion of nonmalarial fevers exceeded 80% in the public health sectors of Kenya, Namibia, Sudan North, Mauritania, Ethiopia, Comoros, Djibouti, Swaziland, and Zimbabwe (Table 1), all of which experience largely low endemicity (*PfPR_2–10_*<5%). It should also be noted, however, that of the five countries with the largest number of nonmalarial fevers, two (Nigeria and DRC) are of predominantly high transmission intensity, together contributing 25.1 million (24%) of the total number of nonmalarial fevers.

### Methodological and Data Limitations

We have used a relatively straightforward approach to generate broad estimates of the number of fever events in African children in 2007, the total seeking care at public health facilities, and the number of those likely to be infected with *P. falciparum*. Various limitations arise from the retrospective assembly of diverse data collected for differing purposes, at different times, and with differing levels of precision.

We have used fevers in young children over the last 14 d reported by their mothers or caretakers as the entry point to estimating treatment needs. The use of the term “fever” can be synonymous with a general state of poor health in some communities rather than a biologically equivalent and clinically relevant increase in body temperature [44]. Despite these limitations fever is often the prompt to seek treatment [45],[46], and most clinical diagnoses and algorithms use fever reported by mother's as the prompt to treat for malaria [5]. The incidence of perceived fever is therefore more important than the precise incidence of clinical malaria when examining the demand for services and commodities but recognizes that not all reports of “fever” are biologically or clinically equivalent fevers [44] and not all fevers are malaria [47],[48].

We assumed that 14-d period prevalence for self-reported fever from national surveys was representative of prevalence across the year. This assumption may not be valid where the causes of fever are strongly seasonal although the temporal correspondence between climatic seasons, malaria, and fevers is complex and spatially heterogeneous across Africa [49]–[52]. Further, the simple multiplication of a 14-d reported incidence to obtain an annual rate can introduce possible bias. If a large proportion of fevers are of relatively long duration, then this approach will tend to overestimate the annualized rate because many episodes in a given 2-wk reporting period actually overlap with preceding or proceeding periods and are therefore effectively double counted. Conversely, however, children experiencing more than one febrile episode during the 2-wk survey period will be recorded as a single fever case, thus leading to an underestimate of total episodes. In the absence of systematically collected and reliable data on child fever durations and frequencies, it is not possible to evaluate these effects more formally.

The assembled national survey data on both fever prevalence and treatment-seeking rates are also limited by their spatial and temporal resolution. We have obtained the most recent available survey from every country and all but seven of the 43 included were collected within 4 y of the target study date of 2007. However, it is well known that malaria prevalence has declined in certain locations in recent years (for example [53]–[55]), and more widespread temporal changes in either fever rates or treatment-seeking behaviours cannot be ruled out where survey data are less recent. Attempting to enumerate any such transitions is beyond the scope of the current study, but we note this fact as an important potential caveat to our 2007 baseline enumeration.

From a geographical perspective, national survey data such as those used in this study can generally be considered representative at the ADMIN1 level, but represent a mean value for each administrative region and therefore mask within-region spatial heterogeneity. Inevitably, therefore, the mean value underestimates the true local values in high areas and overestimates them in low areas. In many regions, systematic differences between urban and rural areas are a major component of this within-region variation, and the procedure implemented in this study to adjust for these urban–rural differentials is likely to mitigate some of the spatial imprecision associated with ADMIN1 data, as is the use of per-pixel delineations of underlying endemicity. Notwithstanding these adjustments, we have chosen to treat the national survey data as fixed, disregarding the effects of sampling error. This decision was driven in part by the difficulty in enumerating sampling errors derived from multistage cluster sampling designs and the unavailability in some cases of required input parameters. More importantly, however, the contribution of this source of uncertainty on the national and continental-level results that form the primary outputs of this study was considered likely to be minimal in comparison to the much larger uncertainty ranges that we present in association with our enumeration of infection rates in presenting fevers in different endemicity strata. In a similar way, we have chosen to treat the underlying modelled *PfPR_2–10_* endemicity surface as fixed. Whilst uncertainty in predicted prevalence is large in some regions, it is only transitions between, rather than within, each of the three defined endemicity strata that would have had a bearing on the results we present here and such an effect is likely to be minimal except around the margins of each risk strata. Again, out focus has been on quantifying the much larger uncertainty associated with the infection rates in fevers at health facilities.

We have used a simple approach to incorporate the variation observed in our assembly of 67 endemicity-stratified studies on infections rates in fevers at clinics. These ranges are based on small sample sizes and precision in our estimates will inevitably increase as additional suitable studies become available. However, we applied lower and upper IQR values universally across Africa and this is likely to yield conservative estimates of our precision since the odds are small that infection rates at all locations display values at the extremes of the observed statistical distributions simultaneously.

We constrain this study to an analysis of fevers in children aged under 5 y. Replicating our approach across all age ranges would require either equivalent continent-wide subnational data on all-age fever prevalence and treatment-seeking behaviour, or reliable biological or empirical models that define age-dependent relationships allowing inference of these variables across the entire age spectrum using our observed results in children. To our knowledge neither are available, which precludes quantitative analysis outside the 0–4-y age range.

### Model Availability and Future Refinement

The geographical information system modelling procedure defined in this study has been packaged as a script (available as Java, Python, or Visual Basic) that can be downloaded freely from the Malaria Atlas Project website (http://www.map.ox.ac.uk/publications). This portable tool has been provided to promote replication of our methods and to facilitate updated analyses as new input data become available. Several future refinements to our model architecture could be envisaged. In particular, as additional data are collected the relationship between transmission intensity and the proportion of fevers attributable to malaria infection in public health facilities can be defined more precisely, potentially modelling a continuous relationship in a Bayesian framework, allowing the inherent uncertainty to be quantified and combined with other uncertainty sources such as those associated with the underlying modelled prevalence.

### Conclusions

Following the introduction of new, effective combination medicines for the treatment of malaria, and ongoing efforts to increase their coverage and availability in public health sectors, concerns have been raised regarding the continued indiscriminate use of antimalarials for all fevers across Africa. We estimate that 57% of the 182 million children presenting with a fever to government supported clinics in Africa do not have a malaria infection, and in some countries that proportion is greater than 90%, highlighting the potential benefits of robust diagnosis to appropriate case management and drug stocking levels. Spatial estimates of fever burdens and the use of health facilities can be combined with relational infection risk models to estimate the degree of parasitemia in fevers presenting to public health facilities. This quantification provides an important baseline comparison of malarial and nonmalarial fevers in different endemicity settings that acts as an alternative to inadequate routine data collection systems and can contribute to ongoing debates about optimum clinical and financial strategies for the introduction of new diagnostics. What these models can never replace is high quality information from public sector services in the form of reliable and complete health information on drug use and patient burdens and whether these patients have peripheral infections. Unfortunately, inadequacies in national health management information systems across Africa [28] are in part a cause of the present imperfections in essential commodity demand and burden estimation.

## Supporting Information

Protocol S1 Implementing urban-rural adjustments to ADMIN1-reported fever prevalence and treatment-seeking rates.
(0.21 MB DOC)

Protocol S2 Systematic literature review on the proportion of febrile children presenting to public health systems that are *P. falciparum* positive.
(0.15 MB DOC)

Protocol S3 Assessing the effect of model configurations on estimated total and *P. falciparum*-positive paediatric fevers attending public health facilities in Africa in 2007.
(0.12 MB DOC)

Table S1 Data assembled to define period prevalence of fever among children 0–4 y at subnational levels from 42 African national sample surveys.
(0.08 MB DOC)

## Abbreviations

ACT: artemisinin-based combination therapy
ADMIN1: first-level administrative unit
GRUMP: Global Rural Urban Mapping Project
IQR: interquartile range
MIS: Malaria Indicator Survey
*Pf*PR_2–10_: *P. falciparum* parasite rate in 2 y up to 10 y olds
RDT: rapid diagnostic test
