# Supplementary material for: Estimating the Number of Paediatric Fevers Associated with Malaria Infection Presenting to Africa's Public Health Sector in 2007
Source: PLoS Med. 2010 Jul 6;7(7):e1000301. doi: 10.1371/journal.pmed.1000301 (PMC2897768; doi:10.1371/journal.pmed.1000301)
Supplement: Protocol S1 — Implementing urban-rural adjustments to ADMIN1-reported fever prevalence and treatment-seeking rates. (0.21 MB DOC) [file pmed.1000301.s001.doc]

**Protocol S1: Implementing urban-rural adjustments to ADMIN1 reported fever prevalence and treatment seeking rates**

Two model inputs were assembled from national surveys using data reported at ADMIN1 level: the annualized prevalence of fevers in children aged 0-4 (i.e. the expected fever rate per child per year), and the proportion of febrile children that sought care from a public health facility. Data from national surveys generally result from a sampling framework designed to provide sufficient precision at the ADMIN1 level and above. However, in this study we wanted to combine the two surveyed variables with a malaria endemicity model which predicted endemicity class membership across a 5×5 km raster grid. in order to retain the spatial precision in this modeled input it was necessary to assign to every 5x5 km pixel a surveyed prevalence and treatment seeking rate value originally presented at ADMIN1 level. Rather than apply ADMIN1 values uniformly to all pixels within each unit boundary, we implemented an urban-rural stratification, seeking to represent a likely source of within-unit heterogeneity in surveyed values. Although reliable urban-rural differences were not available at ADMIN1 level, they were available as national summaries for many countries, and these are summarized in Table S1.1. We devised a Geographic Information System (GIS) procedure that allowed fever prevalence and treatment seeking rates in each pixel to be adjusted to achieve two simultaneous characteristics: (1) that the difference between value in urban and rural pixels within each ADMIN1 unit would match the nationally reported urban-rural ratio and (2) that the population-weighted mean for each ADMIN1 unit would remain unchanged to that originally reported. Figure S1.1 provides a schematic explanation of this procedure for the annualized fever prevalence variable, resulting in a continuous 5×5 km surface in which each pixel contains an urban-rural adjusted prevalence value according to the two conditions stated above. An identical procedure was carried out for the treatment seeking variable.

| Country^1^ | Source | Year | Month | Urban | | | | | Rural | | | | | U-R ratios | |
| --- | --- | --- | --- | --- | --- | --- | --- | --- | --- | --- | --- | --- | --- | --- | --- |
|  |  |  |  | n U5 | n Fever | (%) | n PHF | (%) | n U5 | n Fever | (%) | n PHF | (%) | Fever% | PHF% |
|  |  |  |  |  |  |  |  |  |  |  |  |  |  |  |  |
| Angola | MIS | 2006-7 | Nov-Apr | 951 | 199 | (20.93) | 102 | (51.26) | 1434 | 335 | (23.36) | 127 | (37.91) | 0.90 | 1.35 |
| Benin | DHS | 2006 | Jul-Nov | 5289 | 1381 | (26.11) | 353 | (25.56) | 9393 | 2823 | (30.05) | 733 | (25.97) | 0.87 | 0.98 |
| Burkina Faso | MICS | 2006 | Apr-Jun | 602 | 131 | (21.76) | 8 | (6.11) | 5075 | 920 | (18.13) | 126 | (13.70) | 1.20 | 0.45 |
| Burundi | MICS | 2005 | Sept-Dec | 961 | 158 | (16.44) | 17 | (10.76) | 5973 | 1765 | (29.55) | 340 | (19.26) | 0.56 | 0.56 |
| Cameroon | MICS | 2006 | May-Jun | 2614 | 410 | (15.68) | 68 | (16.59) | 3881 | 744 | (19.17) | 164 | (22.04) | 0.82 | 0.75 |
| Chad | DHS | 2004 | Jul-Dec | 2216 | 678 | (30.60) | 189 | (27.88) | 2710 | 869 | (32.07) | 48 | (5.52) | 0.95 | 5.05 |
| Comoros^2^ | MICS | 2000 | Oct-Dec | 1014 | 82 | (8.10) | 44 | (53.30) | 3856 | 409 | (10.60) | 158 | (38.60) | 0.76 | 1.38 |
| Congo | DHS | 2005 | Jul-Nov | 2678 | 555 | (43.70) | 242 | (43.60) | 1757 | 447 | (25.44) | 183 | (40.94) | 0.81 | 1.07 |
| Côte D'Ivoire | MICS | 2006 | Aug-Oct | 3219 | 722 | (22.43) | 128 | (17.73) | 5385 | 1489 | (27.65) | 180 | (12.09) | 0.81 | 1.47 |
| Djibouti | MIS | 2008-9 | Dec-Feb | 1757 | 309 | (17.60) | 135 | (43.70) | 1265 | 289 | (22.90) | 112 | (38.90) | 0.77 | 1.12 |
| DRC | DHS | 2007 | May-Aug | 3282 | 983 | (30.00) | 266 | (27.06) | 4705 | 1573 | (33.43) | 404 | (25.68) | 0.90 | 1.05 |
| Ethiopia | DHS | 2005 | Apr-Aug | 1275 | 185 | (14.51) | 46 | (24.86) | 7727 | 1402 | (18.14) | 185 | (13.20) | 0.80 | 1.88 |
| Eq. Guinea^3^ | MICS | 2000 | July-Nov | 1161 | 382 | (32.90) | 73 | (19.11) | 1296 | 357 | (27.55) | 67 | (18.77) | 1.19 | 1.02 |
| Gabon | DHS | 2000-1 | Jul-Jan | 2473 | 736 | (29.76) | 204 | (27.72) | 1627 | 427 | (26.24) | 71 | (16.63) | 1.13 | 1.67 |
| Gambia | MICS | 2005-6 | Dec-Mar | 2248 | 201 | (8.94) | 75 | (37.31) | 4393 | 337 | (7.67) | 142 | (42.14) | 1.17 | 0.89 |
| Ghana | MICS | 2006 | Aug-Oct | 1030 | 201 | (19.51) | 68 | (33.83) | 2515 | 595 | (23.66) | 161 | (27.06) | 0.82 | 1.25 |
| Guinea | DHS | 2005 | Feb-Jun | 1242 | 334 | (26.89) | 165 | (49.40) | 4399 | 1457 | (33.12) | 397 | (27.25) | 0.81 | 1.81 |
| Guinea Bissau | MICS | 2006 | May-Jun | 2364 | 312 | (13.20) | 85 | (27.24) | 4206 | 475 | (11.29) | 96 | (20.21) | 1.17 | 1.35 |
| Kenya | MIS | 2007 | Jun-Jul | 1733 | 454 | (26.20) | 135 | (29.80) | 1731 | 457 | (26.40) | 163 | (35.70) | 0.99 | 0.83 |
| Liberia | DHS | 2006-7 | Dec-Apr | 1852 | 554 | (29.91) | 265 | (47.83) | 3453 | 1119 | (32.41) | 398 | (35.57) | 0.92 | 1.34 |
| Madagascar | DHS | 2003-04 | Nov-Mar | 2813 | 532 | (18.91) | 145 | (27.26) | 2275 | 508 | (22.33) | 125 | (24.61) | 0.85 | 1.11 |
| Malawi | MICS | 2006 | Jul-Nov | 2367 | 678 | (28.64) | 101 | (14.95) | 20871 | 7379 | (35.36) | 634 | (8.63) | 0.81 | 1.73 |
| Mali | DHS | 2006 | Apr-Sep | 3791 | 577 | (15.22) | 235 | (40.73) | 8646 | 1517 | (17.55) | 365 | (24.06) | 0.87 | 1.69 |
| Mauritania | DHS | 2003-4 | Aug-Feb | 1327 | 333 | (25.09) | 86 | (25.83) | 1398 | 418 | (29.90) | 115 | (27.51) | 0.84 | 0.94 |
| Mauritania | MICS | 2007 | May-Sept | 3445 | 516 | (14.98) | 91 | (17.64) | 5536 | 929 | (16.78) | 71 | (7.64) | 0.89 | 2.31 |
| Mozambique | DHS | 2003 | Aug-Dec | 3242 | 779 | (24.03) | 486 | (62.39) | 5887 | 1543 | (26.21) | 717 | (46.47) | 0.92 | 1.34 |
| Namibia | DHS | 2006-7 | Oct-Mar | 1855 | 271 | (14.61) | 133 | (49.08) | 3003 | 502 | (16.72) | 261 | (51.99) | 0.87 | 0.94 |
| Niger | DHS | 2006 | Jan-Jun | 2405 | 565 | (23.49) | 199 | (35.22) | 5804 | 1605 | (27.65) | 512 | (31.90) | 0.85 | 1.10 |
| Nigeria | DHS | 2003 | Mar-Aug | 1902 | 510 | (26.81) | 148 | (29.02) | 3284 | 1093 | (33.28) | 238 | (21.77) | 0.81 | 1.33 |
| Rwanda | DHS | 2005 | Feb-Jul | 1582 | 388 | (24.53) | 114 | (29.38) | 6170 | 1613 | (26.14) | 362 | (22.44) | 0.94 | 1.31 |
| ST & P^2^ | MICS | 2000 | Jan-Mar | 1013 | 54 | (5.30) | 16 | (29.80) | 1181 | 48 | (4.10) | 24 | (49.80) | 1.29 | 0.60 |
| Senegal^4^ | MIS | 2008-9 | Dec-Jan | 4949 | 1708 | (16.00) | 673 | (39.40) | 8366 | 2415 | (28.9) | 906 | (37.50) | 1.19 | 1.05 |
| Sierra Leone^5^ | MICS | 2005 | Oct-Nov | 4496 | 1451 | (32.30) | 562 | (38.70) | 1408 | 378 | (26.85) | 149 | (39.50) | 1.20 | 0.98 |
| Somalia | MICS | 2006 | Aug-Sep | 2356 | 338 | (14.30) | 8 | (2.370) | 4017 | 950 | (23.65) | 6 | (0.63) | 0.61 | 3.75 |
| Sudan | SHHS | 2006 | Oct | 4738 | 1521 | (32.10) | 1132 | (74.40) | 4739 | 1706 | (36.00) | 1139 | (78.50) | 0.89 | 0.95 |
| Swaziland | DHS | 2006-7 | Jul-Feb | 638 | 131 | (20.53) | 44 | (33.59) | 1899 | 581 | (30.60) | 268 | (46.13) | 0.67 | 0.73 |
| Tanzania(M)^6^ | AIS-MIS | 2007-8 | Oct-Feb | 812 | 170 | (20.94) | 105 | (61.76) | 4329 | 801 | (18.50) | 390 | (48.69) | 1.13 | 1.27 |
| Tanzania(Z)^6^ | AIS-MIS | 2007-8 | Oct-Feb | 327 | 47 | (14.37) | 34 | (72.34) | 1563 | 182 | (11.64) | 121 | (66.48) | 1.23 | 1.09 |
| Togo^5^ | MICS | 2006 | May-June | 1224 | 212 | (17.32) | 34 | (16.00) | 2930 | 554 | (18.91) | 85 | (15.30) | 0.92 | 1.05 |
| Uganda | DHS | 2006 | May-Oct | 847 | 199 | (23.49) | 55 | (27.64) | 6746 | 2892 | (42.87) | 869 | (30.05) | 0.55 | 0.92 |
| Zambia | DHS | 2007 | April-Oct | 1873 | 318 | (16.98) | 217 | (68.24) | 3971 | 716 | (18.03) | 385 | (53.77) | 0.94 | 1.27 |
| Zimbabwe | DHS | 2005-6 | Aug-Mar | 1259 | 85 | (6.75) | 25 | (29.41) | 3616 | 306 | (8.46) | 98 | (32.03) | 0.80 | 0.92 |
|  |  |  |  |  |  |  |  |  |  |  |  |  |  |  |  |
| 1. Data not available for Eritrea, South Africa, Botswana, Cape Verde and Central African Republic; 2. Used treatment seeking for acute respiratory infection; 3. Used treatment seeking for cough; 4. Used MIS Nov-Dec 2006 for proportion accessing PHF; 5. Used treatment seeking for pneumonia; 6. Used separate values for Tanzania mainland (M) and Zanzibar (Z). | | | | | | | | | | | | | | | |

**Table S1.1 National-level differences in reported period fever prevalence and treatment seeking rates between urban and rural areas.** For each country the number (n U5) of 0-4 yr olds included in national surveys and reporting fever in the 14 preceding days (n Fever) is given along with the number attending a public health facility (n PHF) for both urban and rural settings. Also shown are the ratios of these urban *vs.* rural values. Democratic Republic of Congo (DRC); São Tomé and Principe (ST & P).


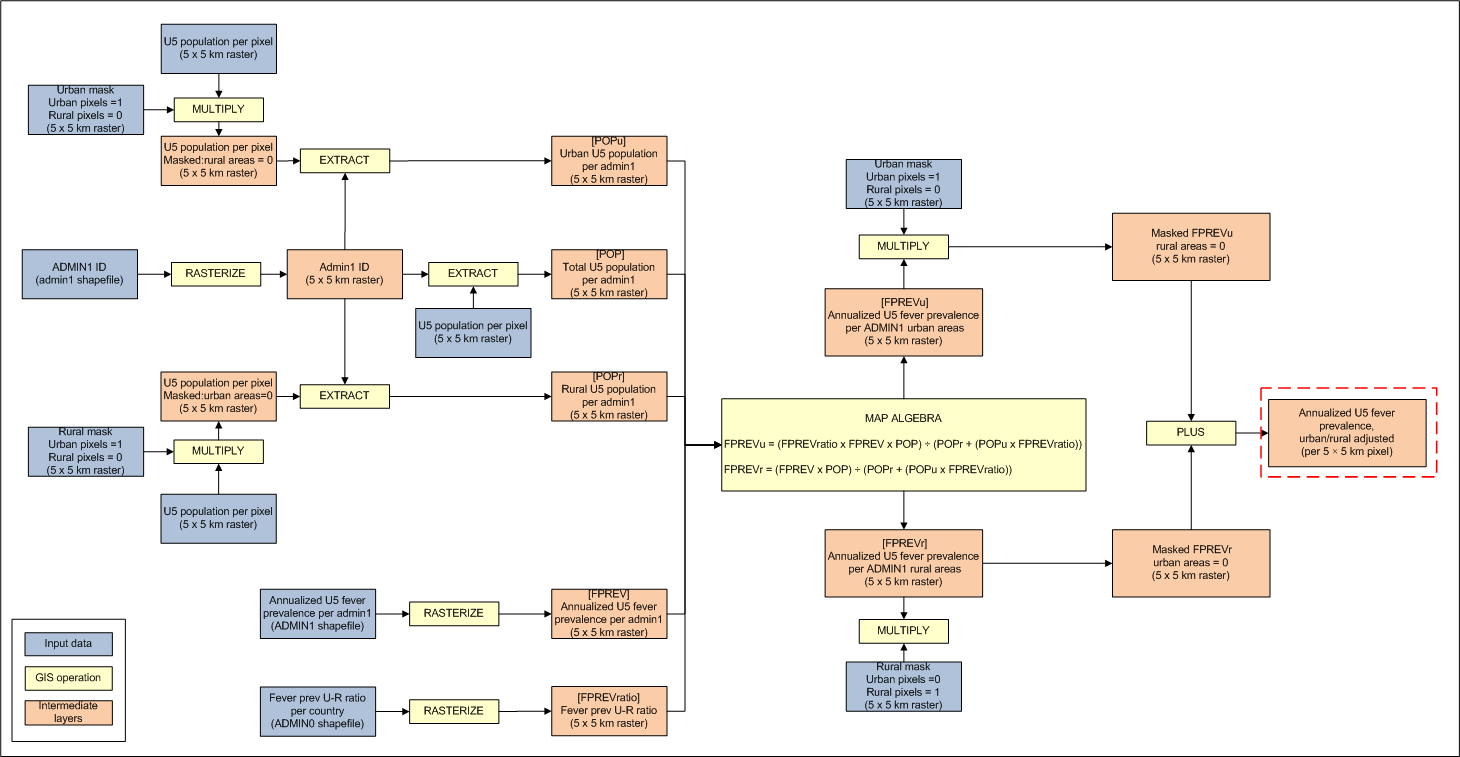


**Figure S1.1. GIS procedure for implementing urban-rural adjustments to ADMIN1-level annualized fever prevalence rates.** Blue boxes describe input data; yellow boxes denote GIS operations; orange boxes denote interim output. The dashed red box highlights the end point of this procedure, and this box matches exactly a component of the simplified flowchart shown in the main text. U5 = children aged under five years old.
