# Supplementary material for: Estimating the Number of Paediatric Fevers Associated with Malaria Infection Presenting to Africa's Public Health Sector in 2007
Source: PLoS Med. 2010 Jul 6;7(7):e1000301. doi: 10.1371/journal.pmed.1000301 (PMC2897768; doi:10.1371/journal.pmed.1000301)
Supplement: Protocol S2 — Systematic literature review on the proportion of febrile children presenting to public health systems that are P. falciparum positive. (0.15 MB DOC) [file pmed.1000301.s002.doc]

**Protocol S2: Systematic literature review on the proportion of febrile children presenting to public health systems that are *P. falciparum* positive.**

We performed a PubMed search using the Medical Subject Headings (MeSH) terms “malaria diagnosis Africa” on titles, abstract and full text and selected all publications since 2000. This generated 1,802 titles and abstracts which were reviewed for possible information on the proportion of febrile children at clinic who were confirmed to have evidence of *P. falciparum* infection. 168 potentially suitable publications were identified for review. 19 papers could not be sourced from national libraries or on-line resources. 99 reports were excluded because: a) they lacked sufficient detail on the age structure of investigated patients or were among adults only making a selection of paediatric populations impossible (n=33); b) selection of drug trial participants did not have enough detail on those excluded because of slide negative results (n=22); c) reasons for screening were unclear (n=9); d) final parasitological diagnoses were using routine laboratory services or RDT, rather than carried out explicitly for the study in question, and therefore of unknown provenance (n=11); and e) were simply not related to the parameters of interest (n=24). 50 publications included information on patients screened for infection at out-patient clinics as part of surveillance, clinical audits, drug trials or evaluations of diagnostic tests that could be used in the analysis. Finally we accessed information from clinical sites run by MSF-Spain in Somalia, Central African Republic and Sierra Leone to seek additional information from clinical audits and surveillance performed in these countries (resulting in an additional four study sites in Somalia). Selection criteria for detailed microscopy varied between studies ranging from definitions of raised body temperature and/or histories of fever in the last 24, 48 and 72 hours to unspecified criteria such as “suspected” or “presumed” malaria. As such the denominator inclusions varied between studies and were hard to control for. In addition the age ranges included in the series varied between studies; for most drug trials children were aged 6 months up to their fifth birthday and across other types of investigation studies included children up to 15 years of age.

**Table S2**.1 (part 1 of 2). Study descriptive for health facility surveys that described infection prevalence in “suspected” malaria cases

| **Ref** | **Country** | **Places** | **^1^Type** | **Year** | **Entry criteria** | **^2^Age (yr)** | **^3^Exam.** | **^4^­*Pf* +ve (%)** | **^5^*Pf*PR_2-10_(%)** | **^6^PR class** |
| --- | --- | --- | --- | --- | --- | --- | --- | --- | --- | --- |
| 1 | Angola | Luanda | 1 | 2008 | Ho fever in 24hrs +/or AX T ≥ 37.5^o^C no signs severe M | 0-4 | 494 | 2 | 10 | 2 |
| 2 | Angola | Caala Town | 2 | 2004 | Suspected malaria | 0.5-4 | 742 | 23 | 32 | 3 |
| 3 | Benin | Cotonou | 1 | 2003 | Ho fever in last 36 hrs +/or Ax T ≥ 37.5^o^C | 0-5 | 131 | 4 | 34 | 3 |
| 4 | Burkina Faso | Ouagadougou | 1 | 2002 | Ho fever in last 36 hrs +/or Ax T ≥ 37.5^o^C | 0-4 | 232 | 22 | 60 | 4 |
| 5 | Burkina Faso | Bobo Dioulasso | 4 | ?2006 | Ax T ≥ 37.5^o^C no severe signs | 0.5-15 | 2141 | 47 | 52 | 4 |
| 6 | Cameroun | Yaounde | 4 | 2007-08 | Ho fever in last 24 hrs +/or Ax T ≥ 37.5^o^C | 0-9 | 313 | 41 | 54 | 4 |
| 7 | Chad | N'Jamena | 3 | 2002-03 | Diagnosed with presumed malaria | 0-4 | 160 | 19 | 15 | 3 |
| 8 | Congo | Madibu, Brazaville | 1 | 2003-06 | Febrile | 0-4 | 558 | 47 | 39 | 3 |
| 8 | Congo | Tenrikyo, Brazaville | 1 | 2003-06 | Febrile | 0-4 | 7365 | 24 | 48 | 4 |
| 9 | Congo | Kindomba | 2 | 2004 | Ax T ≥ 37.5^o^C or Ho fever last 24 hrs | 0.5-4 | 526 | 94 | 55 | 4 |
| 10 | Côte D'Ivoire | Yopongon, Abidjan | 1 | 2002 | Ho fever in last 36 hrs +/or Ax T ≥ 37.5^o^C | 0-4 | 220 | 36 | 68 | 4 |
| 11 | Ethiopia | Alaba Kulito | 1 | 2007 | Febrile with signs of malaria | 1-4 | 171 | 5 | 14 | 3 |
| 12 | Gabon | Libreville | 1 | 2001-02 | Ho of fever in last 24 hrs | 0-10 | 8195 | 43 | 37 | 3 |
| 13 | Gabon | Oyem | 2 | 2005 | Ax T ≥ 37.5^o^C and no of severe signs or other causes | 0.5-4 | 632 | 48 | 65 | 4 |
| 14 | Ghana | Kintampo | 2 | 2005-06 | Ho fever +/or Ax T ≥ 37.5oC | 0.5-10 | 1717 | 59 | 46 | 4 |
| 15 | Ghana | Navrongo | 2 | ?2003 | Suspected of malaria, Ho of fever | 0-4 | 922 | 80 | 63 | 4 |
| 16 | Ghana | Navrongo | 2 | 2005-06 | Suspected malaria - acute febrile illness | 0.5-9 | 638 | 56 | 63 | 4 |
| 17 | Kenya | Mbita | 3 | 2003-05 | Ho of fever on day of presentation | 0-4 | 2496 | 60 | 20 | 3 |
| 18 | Kenya | Mbita | 4 | 2007 | Clinical suspicion, Ho fever in last 48 hrs +/or Ax T ≥ 37.5^o^C | 0.5-12 | 650 | 17 | 20 | 3 |
| 19 | Kenya | Pinglilikani | 3 | 2003-09 | Clinical history suggestive of malaria | 0-4 | 23871 | 35 | 22 | 3 |
| 20 | Kenya | Chulaiambo | 2 | 2007 | Ax T ≥ 37.5^o^C +/or Ho fever in last 10 days, excluded severe M | 0.5-4 | 1321 | 28 | 33 | 3 |
| 21 | Kenya | Kimbimbi | 4 | 2005 | Suspected of malaria Ho fever last 24 hours +/or Ax T ≥ 37.5^o^C | 0.5-12 | 184 | 33 | 1 | 2 |
| 22 | Mali | Missidoougou | 4 | 2006 | Suspected malaria on basis of fever | 0-5 | 158 | 92 | 66 | 4 |
| 23 | Mozambique | Mocuba | 1 | 2004 | Clinical diagnosis of malaria and not a follow-up visit | 0.5-13 | 366 | 77 | 40 | 4 |
| 24 | Mozambique | Hoke | 4 | 2000 | "clinical malaria" + fever in last 36 hrs +/or Ax T ≥ 37.5^o^C | 0-14 | 130 | 75 | 54 | 4 |
| 25 | Mozambique | Manhica | 3 | 2003-05 | Ho fever in last 24 hours +/or Ax T ≥ 37.5^o^C | 0-14 | 28963 | 37 | 47 | 4 |
| 26 | Nigeria | Ibadan | 2 | 2004 | Symptoms compatible with acute *P. falciparum* malaria | 0-12 | 412 | 61 | 51 | 4 |
| 27 | Nigeria | Ibadan | 2 | 1996-04 | Symptoms compatible with acute *P falciparum* malaria | 0-13 | 1643 | 51 | 51 | 4 |
| 28 | Nigeria | Jos | 2 | 2001 | Ho of fever | 0-14 | 146 | 76 | 56 | 4 |
| 29 | Senegal | Thies and Mbour | 5 | 2004 | Febrile with signs of malaria excluding signs of severe malaria | 2-10 | 289 | 60 | 22 | 3 |
| 30 | Sierra Leone | Various | 2 | 2002-03 | Suspected of malaria | 0.5-4 | 2579 | 62 | 52 | 4 |
| 31 | Somalia | Buulo sheekh | 3 | 2007 | Clinical suspicion of malaria | 0-4 | 2983 | 0 | 5 | 2 |
| 31 | Somalia | Burane | 3 | 2007 | Clinical suspicion of malaria | 0-4 | 812 | 0 | 3 | 2 |
| 31 | Somalia | Kulmis | 3 | 2007 | Clinical suspicion of malaria | 0-4 | 1741 | 1 | 3 | 2 |
| 31 | Somalia | Mahaday | 3 | 2007 | Clinical suspicion of malaria | 0-4 | 829 | 0 | 2 | 2 |
| 32 | Sudan | Limun | 2 | 2003 | Ho fever | 0.5-4 | 307 | 65 | 12 | 3 |
| 33 | Sudan | Khartoum Centre | 3 | 2005 | Clinical suspicion of malaria | 0-9 | 1650 | 1 | 0 | 1 |
| 34 | Sudan | Khartoum Centre | 3 | 2002 | History of fever in last 72 hours | 0.2-16 | 655 | 12 | 0 | 1 |
| 35 | Tanzania | Chake/Tosamagna | 4 | 2007 | Ax T ≥ 37.5^o^C +/or Ho fever in last 10 days, no severe M, diseases or AM use | 0-4 | 92 | 13 | 1 | 2 |
| 36 | Tanzania | Chalinze | 5 | 2003-04 | H^o^ fever in last 48 hrs +/or Ax T ≥ 37.5^o^C, plus consent to return in 7/7 | 0-4 | 200 | 30 | 26 | 3 |
| 51 | Tanzania | Dar es Salaam | 1 | 2003 | Ho fever in last 36 hrs +/or Ax T > 37.5^o^C | 0-4 | 312 | 5 | 4 | 2 |
| **Ref** | **Country** | **Places** | **^1^Type** | **Year** | **^2^Entry criteria** | **^3^Age (yr)** | **^4^Exam.** | **^5^­*Pf* +ve (%)** | **^6^*Pf*PR_2-10_(%)** | **^7^PR class** |
| 21 | Tanzania | Dar es Salaam | 4 | 2005 | Suspected malaria Ho fever last 24 hours and/or Ax T ≥ 37.5^o^C | 0.5-12 | 154 | 2 | 4 | 2 |
| 37 | Tanzania | Kibaha | 4 | 2000 | Ho fever in last 4/7 +/or Ax T ≥ 37.5^o^C and/or palmor pallor | 0.1-4 | 395 | 70 | 13 | 3 |
| 36 | Tanzania | Kikongo | 5 | 2003-04 | Ho fever in last 48 hrs +/or Ax T ≥ 37.5^o^C, plus consent to return in 7/7 | 0-4 | 194 | 35 | 17 | 3 |
| 36 | Tanzania | Mbwewe | 5 | 2003-04 | Ho fever in last 48 hrs +/or Ax T ≥ 37.5^o^C, plus consent to return in 7/7 | 0-4 | 191 | 46 | 22 | 3 |
| 36 | Tanzania | Mwanabwito | 5 | 2003-04 | Ho fever in last 48 hrs +/or Ax T ≥ 37.5 ^o^C, plus consent to return in 7/7 | 0-4 | 176 | 36 | 12 | 3 |
| 38 | Tanzania | Rufiji | 1 | 2004 | Clinical Dx | 0-4 |  | 43 | 25 | 3 |
| 39 | Tanzania | Makorora | 4 | 2005 | Febrile with signs of malaria | 0-4 | 252 | 23 | 7 | 3 |
| 36 | Tanzania | Ubena | 5 | 2003-04 | Ho fever in last 48 hrs +/or Ax T ≥ 37.5 ^o^C, plus consent to return in 7/7 | 0-4 | 176 | 47 | 28 | 3 |
| 40 | Tanzania | Magoda & Mpapaya | 2 | ?2003 | ? Febrile children screened at mobile clinic | 0.5-4 | 269 | 49 | 17 | 3 |
| 41 | Tanzania | Same | 3 | 2001-05 | Clinical diagnosis of malaria | 0-14 | 30087 | 11 | 3 | 2 |
| 42 | Tanzania | Muyuni, Uzini etc | 4 | 2005 | Fever clinical diagnosis of malaria | 0-4 | 1047 | 36 | 1 | 2 |
| 43 | Uganda | Apac | 4 | 2006-07 | OPD referred for microscopy suspected clin as malaria vs expert M | 0-4 | 793 | 85 | 37 | 3 |
| 43 | Uganda | Arua | 4 | 2006-07 | OPD referred for microscopy suspected clin as malaria vs expert M | 0-4 | 350 | 63 | 41 | 4 |
| 43 | Uganda | Jinja | 4 | 2006-07 | OPD referred for microscopy suspected clin as malaria vs expert M | 0-4 | 354 | 33 | 26 | 3 |
| 43 | Uganda | Kabale | 4 | 2006-07 | OPD referred for microscopy suspected clin as malaria vs expert M | 0-4 | 278 | 4 | 0 | 1 |
| 44 | Uganda | Kamwezi | 1 | 2001-03 | Ho of fever | 0-4 | 62 | 40 | 26 | 3 |
| 43 | Uganda | Kanungu | 4 | 2006-07 | OPD referred for microscopy suspected clin as malaria vs expert M | 0-4 | 510 | 47 | 28 | 3 |
| 43 | Uganda | Kyenjojo | 4 | 2006-07 | OPD referred for microscopy suspected clin as malaria vs expert M | 0-4 | 335 | 62 | 46 | 4 |
| 45 | Uganda | Mbarara | 4 | 2000-01 | Clinical symptoms of malaria | 0-4 | 315 | 66 | 19 | 3 |
| 46 | Uganda | Mbarara | 4 | 2005 | Symptoms suggestive of malaria weighing >5kg no severe signs | 0-4 | 239 | 54 | 19 | 3 |
| 47 | Uganda | Mubende & Jinja | 1 | 2007 | Ho of fever | 0-4 | 288 | 37 | 36 | 3 |
| 48 | Uganda | Mulago, Kampala | 4 | 2004-05 | Tympanic T ≥ 38 ^o^C +/or Ho fever in last 24hrs | 1-10 | 862 | 33 | 19 | 3 |
| 49 | Uganda | Mulago, Kampala | 3 | 2005-08 | Tympanic T ≥ 38 ^o^C +/or Ho fever in last 24hrs | 1-10 | 3158 | 29 | 19 | 3 |
| 50 | Uganda | Soroti | 4 | 2006-07 | Ho fever in last 24 hrs +/or Ax T ≥ 37.5 ^o^C; no other cause, no danger signs | 0.5-4 | 165 | 62 | 24 | 3 |
| 43 | Uganda | Tororo | 4 | 2006-07 | OPD referred for microscopy suspected as malaria vs expert M | 0-4 | 540 | 59 | 35 | 3 |
| 47 | Uganda | Tororo & Apac | 1 | 2007 | Ho of fever | 0-4 | 206 | 54 | 48 | 4 |
|  |  |  |  |  |  |  |  |  |  |  |
| 1. Type of study included. Categories are: 1 = studies undertaken as part of a clinical audit; 2 = drug trials; 3 = surveillance; 4 = RDT evaluations; and 5 = other studies of diagnostic algorithms or patient adherence.  2. Ho = History of; Ax T = Axillary Temperature; M = Malaria; AM = Antimalarial; Dx = Diagnosis; OPD = Out-Patient Department.  3. Age range of study participants.  4. Number of study participants examined.  5. Number of study participants positive for *Plasmodium falciparum*.  6. Model-based geostatistical estimate of *Plasmodium falciparum* prevalence in 2-10 yr olds.  7. Predicted endemicity class: 1 = unstable transmission; 2=*Pf*PR_2-10_ ≤5%; 3 = *Pf*PR_2-10_ >5% - <40%; 4= *Pf*PR_2-10_ ≥40%. | | | | | | | | | | |

**Table S2.1 (part 2 of 2). Study descriptive for health facility surveys that described infection prevalence in “suspected” malaria cases**

**References**

[1] Thwing JI, Mihigo J, Fernandes AP, Saute F, Ferreira C, Fortes F, de Oliveira AM, Newman RD (2009). How much malaria occurs in urban Luanda, Angola? A health facility-based assessment. *American Journal of Tropical Medicine & Hygiene*, **80**: 487-491.

[2] Guthmann JP, Cohuet S, Rigutto C, Fortes F, Saraiva N, Kiguli J, Kyomuhendo J, Francis M, Noël F, Mulemba M, Balkan S (2006). High efficacy of two artemisinin-based combinations (artesunate + amodiaquine and artemether + lumefantrine) in Caala, Central Angola. *American Journal of Tropical Medicine & Hygiene*, **75**: 143-145.

[3] Wang SJ, Lengeler C, Smith TA, Vounatsou P, Akogbeto M, Tanner M (2006). Rapid Urban Malaria Appraisal (RUMA) IV: epidemiology of urban malaria in Cotonou (Benin). *Malaria Journal*, **5**: e45.

[4] Wang SJ, Lengeler C, Smith TA, Vounatsou P, Diadie DA, Pritroipa X, Convelbo N, Kientga M, Tanner M (2005). Rapid urban malaria appraisal (RUMA) I: epidemiology of urban malaria in Ouagadougou. *Malaria Journal*, **4**: e43.

[5] Bisoffi Z, Sirima BS, Angheben A, Lodesani C, Gobbi F, Tinto H, Van den Ende J (2009a). Rapid malaria diagnostic tests vs. clinical management of malaria in rural Burkina Faso: safety and effect on clinical decisions. A randomized trial. *Tropical Medicine & International Health*, **14**: 491-498.

[6] Sayang C, Soula G, Tahar R, Basco LK, Gazin P, Moyou-Somo R, Delmont J (2009). Use of a histidine-rich protein 2-based rapid diagnostic test for malaria by health personnel during routine consultation of febrile outpatients in a peripheral health facility in Yaoundé Cameroon. *American Journal of Tropical Medicine & Hygiene,* **81**: 343-347.

[7] Othnigué N, Wyss K, Tanner M, Genton B (2006). Urban malaria in the Sahel: prevalence and seasonality of presumptive malaria and parasitaemia at primary care level in Chad. *Tropical Medicine & International Health*, **11**: 204-210.

[8] Ndounga M, Casimiro PN, Miakassissa-Mpassi V, Loumouamou D, Ntoumi F, Basco LK (2008). Malaria in health centres in the southern districts of Brazzaville, Congo. *Bulletin Societie de Pathologie Exotique*, **101**: 329 335.

[9] van den Broek I, Kitz C, Al Attas S, Libama F, Balasegaram M, Guthmann JP (2006). Efficacy of three artemisinin combination therapies for the treatment of uncomplicated *Plasmodium falciparum* malaria in the Republic of Congo. *Malaria Journal*, **5**: e113.

[10] Wang SJ, Lengeler C, Smith TA, Vounatsou P, Cisse G, Tanner M (2006). Rapid Urban Malaria Appraisal (RUMA) III: epidemiology of urban malaria in the municipality of Yopougon (Abidjan). *Malaria Journal,* **5**: e29.

[11] Degarege A, Animut A, Legesse M, Erko B (2009). Malaria severity status in patients with soil-transmitted helminth infections. *Acta Tropica,* **112**: 8-11.

[12] Bouyou-Akotet M, Dzeing-Ella A, Kendjo E, Etoughe D, Ngoungou EB, Planche T, Koko J, Kombila M (2009). Impact of *Plasmodium falciparum* infection on the frequency of moderate to severe anaemia in children below 10 years of age in Gabon. *Malaria Journal,* **8**: e166.

[13] Nsimba B, Guiyedi V, Mabika-Mamfoumbi M, Mourou-Mbina JR, Ngoungou E, Bouyou-Akotet M, Loembet R, Durand R, Le Bras J, Kombila M (2008). sulphadoxine/pyrimethamine versus amodiaquine for treating uncomplicated childhood malaria in Gabon: a randomized trial to guide national policy. *Malaria Journal*, **7**: e31.

[14] Owusu-Agyei S, Asante KP, Owusu R, Adjuik M, Amenga-Etego S, Dosoo DK, Gyapong J, Greenwood B, Chandramohan D (2008). An open label, randomised trial of artesunate+amodiaquine, artesunate+chlorproguanil-dapsone and artemether-lumefantrine for the treatment of uncomplicated malaria. *PLoS One*, **3**: e2530.

[15] Oduro AR, Anyorigiya T, Hodgson A, Ansah P, Anto F, Ansah NA, Atuguba F, Mumuni G, Amankwa J (2005). A randomized comparative study of chloroquine, amodiaquine and sulphadoxine-pyrimethamine for the treatment of uncomplicated malaria in Ghana. *Tropical Medicine & International Health*, **10**: 279-284.

[16] Oduro AR, Anyorigiya T, Anto F, Amenga-Etego L, Ansah NA, Atobrah P, Ansah P, Koram K (2008). A randomized, comparative study of supervised and unsupervised artesunate- amodiaquine, for the treatment of uncomplicated malaria in Ghana. *Annals of Tropical Medicine & Parasitology*,**102**: 565-576.

[17] Fillinger (pers. comm.).

[18] Mens PF, van Amerongen A, Sawa P, Kager PA, Schallig HD (2008). Molecular diagnosis of malaria in the field: development of a novel 1-step nucleic acid lateral flow immunoassay for the detection of all 4 human *Plasmodium* spp. and its evaluation in Mbita, Kenya. *Diagnostic Microbiology and Infectious Diseases*, **61**: 421-427.

[19] Borrmann (pers. comm.).

[20] Juma EA, Obonyo CO, Akhwale WS, Ogutu BR (2008). A randomized, open-label, comparative efficacy trial of artemether-lumefantrine suspension versus artemether-lumefantrine tablets for treatment of uncomplicated *Plasmodium falciparum* malaria in children in western Kenya. *Malaria Journal*, **7**: e262.

[21] Mens P, Spieker N, Omar S, Heijnen M, Schallig H, Kager PA (2007). Is molecular biology the best alternative for diagnosis of malaria to microscopy? A comparison between microscopy, antigen detection and molecular tests in rural Kenya and urban Tanzania. *Tropical Medicine & International Health*, **12**: 238-244.

[22] Willcox ML, Sanogo F, Graz B, Forster M, Dakouo F, Sidibe O, Falquet J, Giani S, Diakite C, Diallo D (2009). Rapid diagnostic tests for the home-based management of malaria, in a high-transmission area. *Annals of Tropical Medicine & Parasitololgy*, **103**: 3-16.

[23] Hume JC, Barnish G, Mangal T, Armázio L, Streat E, Bates I (2008). Household cost of malaria over-diagnosis in rural Mozambique. *Malaria Journal*, **7**: e33.

[24] Hashizume M, Kondo H, Murakami T, Kodama M, Nakahara S, Lucas MES, Wakai S (2006). Use of rapid diagnostic tests for malaria in an emergency situation after the flood disaster in Mozambique. *Public Health*, **120**: 444–447.

[25] Guinovart C, Bassat Q, Sigaúque B, Aide P, Sacarlal J, Nhampossa T, Bardají A, Nhacolo A, Macete E, Mandomando I, Aponte JJ, Menéndez C, Alonso PL (2008). Malaria in rural Mozambique. Part I: Children attending the outpatient clinic. *Malaria Journal*, **7**: e36.

[26] Sowunmi A, Fehintola FA, Adedeji AA, Gbotosho GO, Tambo E, Fateye BA, Happi TC, Oduola AM (2005). Open randomized study of artesunate-amodiaquine vs. chloroquine-pyrimethamine-sulfadoxine for the treatment of uncomplicated *Plasmodium falciparum* malaria in Nigerian children. *Tropical Medicine & International Health*, **10**: 1161-1170.

[27] Sowunmi A, Fateye BA, Adedeji AA, Gbotosho GO, Happi TC, Bamgboye AE, Bolaji OM, Oduola AM (2006). Predictors of the failure of treatment with pyrimethamine-sulfadoxine in children with uncomplicated falciparum malaria. *Acta Tropica*, **98**: 6-14.

[28] Pitmang SL, Thacher TD, Madaki JK, Egah DZ, Fischer PR (2005). Comparison of sulfadoxine-pyrimethamine with and without chloroquine for uncomplicated malaria in Nigeria. *American Journal of Tropical Medicine & Hygiene*, **72**: 263-266.

[29] Souares A, Lalou R, Sene I, Sow D, Le Hesran JY (2009). Factors related to compliance to anti-malarial drug combination: example of amodiaquine/sulphadoxine-pyrimethamine among children in rural Senegal. *Malaria Journal,* **8**: e118.

[30] Checchi F, Roddy P, Kamara S, Williams A, Morineau G, Wurie AR, Hora B, Lamotte N, Baerwaldt T, Heinzelmann A, Danks A, Pinoges L, Oloo A, Durand R, Ranford-Cartwright L, Smet M (2005). Sierra Leone Antimalarial Efficacy Study Collaboration. Evidence basis for antimalarial policy change in Sierra Leone: five in vivo efficacy studies of chloroquine, sulphadoxine-pyrimethamine and amodiaquine. *Tropical Medicine & International Health*, **10**: 146-153.

[31] MSF Spain (pers. comm.).

[32] Hamour S, Melaku Y, Keus K, Wambugu J, Atkin A, Montgomery J, Ford N, Hook C, Checchi F (2005). Malaria in the Nuba Mountains of Sudan: baseline genotypic resistance and efficacy of the artesunate plus sulfadoxine—pyrimethamine and artesunate plus amodiaquine combinations. *Transactions of the Royal Society of Tropical Medicine & Hygiene,* **99**: 548-554.

[33] A-Elgayoum SM, El-Feki Ael-K, Mahgoub BA, El-Rayah el-A, Giha HA (2009). Malaria over-diagnosis and burden of malaria misdiagnosis in the suburbs of central Sudan: special emphasis on artemisinin-based combination therapy era. *Diagnostic Microbiology and Infectious Diseases*, **64**: 20-26.

[34] Malik EM, Eltahir HG, Ahmed ES (2005). Clinical and laboratory aspects of malaria among children with fever in a low transmission area of Sudan. *Eastern Mediterranean Health Journal*, **11**: 753-761.

[35] Nicastri E, Bevilacqua N, Schepisi MS, Paglia MG, Meschi S, Ame SM, Mohamed JA, Mangi S, Fumakule R, Di Caro A, Capobianchi MR, Kitua A (2009). Accuracy of malaria diagnosis by microscopy, rapid diagnostic test, and PCR methods and evidence of antimalarial over prescription in non-severe febrile patients in two Tanzanian hospitals. *American Journal of Tropical Medicine & Hygiene*, **80**: 712-71.

[36] Ngasala B, Mubi M, Warsame M, Petzold MG, Massele AY, Gustafsson LL, Tomson G, Premji Z, Bjorkman A (2008). Impact of training in clinical and microscopy diagnosis of childhood malaria on antimalarial drug prescription and health outcome at primary health care level in Tanzania: a randomized controlled trial. *Malaria Journal*, **7**: e199.

[37] Tarimo DS, Minjas JN, Bygbjerg IC (2001) Malaria diagnosis and treatment under the strategy of the integrated management of childhood illness (IMCI): relevance of laboratory support from the rapid immunochromatographic tests of ICT Malaria P.f/P.v and OptiMal. Source: *Annals of Tropical Medicine and Parasitology*, 95: 437-444.

[38] Kachur PS, Schulden J, Goodman CA, Kassala H, Elling BF, Khatib RA, Causer LM, Mkikima S, Abdulla S, Peter B. Bloland BP (2006). Prevalence of malaria parasitemia among clients seeking treatment for fever or malaria at drug stores in rural Tanzania 2004. *Tropical Medicine & International Health*, **11**: 441- 451.

[39] Kamugisha ML, Msangeni H, Beale E, Malecela EK, Akida J, Ishengoma DRS, Lemnge MM (2008). Paracheck Pf® compared with microscopy for diagnosis of *Plasmodium falciparum* malaria among children in Tanga City, north-eastern Tanzania. *Tanzanian Journal of Health Research,* **10**: 14-19.

[40] Lemnge M, Alifrangis M, Kafuye MY, Segeja MD, Gesase S, Minja D, Massaga JJ, Rønn AM, Bygbjerg IC (2006). High reinfection rate and treatment failures in children treated with amodiaquine for falciparum malaria in Muheza villages, Northeastern Tanzania. *American Journal of Tropical Medicine & Hygiene*, **75**: 188-193.

[41] Masika PM, Semarundu WJ, Urassa R, Mosha J, Chandramohan D, Gosling RD (2006). Over-diagnosis of malaria is not a lost cause. *Malaria Journal*, **5**: e120.

[42] Msellem MI, Martensson A, Rotllant G, Bhattarai A, Stromberg J, Kahigwa E, Garcia M, Petzold M, Olumese P, Ali A, Bjorkman A (2009). Influence of rapid malaria diagnostic tests on treatment and health outcome in fever patients, Zanzibar – a crossover validation study. *PLoS Medicine,* **6:** e1000070.

[43] Hopkins H, Bebell L, Kambale W, Dokomajilar C, Rosenthal PJ, Dorsey G (2008). Rapid diagnostic tests for malaria at sites of varying transmission intensity in Uganda. *Journal of Infectious Disease*, **197**: 510-518.

[44] Ndyomugyenyi R, Magnussen P, Clarke S (2007). Diagnosis and treatment of malaria in peripheral health facilities in Uganda: findings from an area of low transmission in south-western Uganda. *Malaria Journal,* **6**: e39.

[45] Guthmann JP, Ruiz A, Priotto G, Kiguli J, Bonte L, Legros D (2002). Validity, reliability and ease of use in the field of five rapid tests for the diagnosis of *Plasmodium falciparum* malaria in Uganda. *Transactions of the Royal Society of Tropical Medicine & Hygiene,* **96**: 254-257.

[46] Fogg C, Twesigye R, Batwala V, Piola P, Nabasumba C, Kiguli J, Mutebi F, Hook C, Guillerm M, Moody A, Guthmann JP (2008). Assessment of three new parasite lactate dehydrogenase (pan-pLDH) tests for diagnosis of uncomplicated malaria. *Transactions of Royal Society of Tropical Medicine & Hygiene*, **102**: 25-31.

[47] Nankabirwa J, Zurovac D, Njogu JN, Rwakimari JB, Counihan H, Snow RW, Tibenderana JK (2009). Malaria misdiagnosis in Uganda – implications for policy change. *Malaria Journal*, **8**: e66.

[48] Hopkins H, Kambale W, Kamya MR, Staedke SG, Dorsey G, Rosenthal PJ (2007). Comparison of hrp2- and pldh-based rapid diagnostic tests for malaria with longitudinal follow-up in Kampala, Uganda. *American Journal of Tropical Medicine & Hygiene,* **76**: 1092–1097.

[49] Jensen TP, Bukirwa H, Njama-Meya D, Francis D, Kamya MR, Rosenthal PJ, Dorsey G (2009). Use of the slide positivity rate to estimate changes in malaria incidence in a cohort of Ugandan children. *Malaria Journal,* **8**: e213.

[50] Kyabayinze DJ, Tibenderana JK, Odong GW, Rwakimari JB, Counihan H (2008). Operational accuracy and comparative persistent antigenicity of HRP2 rapid diagnostic tests for *Plasmodium falciparum* malaria in a hyperendemic region of Uganda. *Malaria Journal*, **7**: e221.

[51] Wang SJ, Lengeler C, Mtasiwa D, Mshana T, Manane L, Maro G, Tanner M (2006). Rapid Urban Malaria Appraisal (RUMA) II: epidemiology of urban malaria in Dar es Salaam (Tanzania). *Malaria Journal*, **5**: e28.
