# Supplementary material for: Estimating the Number of Paediatric Fevers Associated with Malaria Infection Presenting to Africa's Public Health Sector in 2007
Source: PLoS Med. 2010 Jul 6;7(7):e1000301. doi: 10.1371/journal.pmed.1000301 (PMC2897768; doi:10.1371/journal.pmed.1000301)
Supplement: Protocol S3 — Assessing the effect of model configurations on estimated total and P. falciparum-positive paediatric fevers attending public health facilities in Africa in 2007. (0.12 MB DOC) [file pmed.1000301.s003.doc]

**Protocol S3: Assessing the effect of model configurations on estimated total and *P. falciparum* positive paediatric fevers attending public health facilities in Africa in 2007**

A simple sensitivity analysis was performed to assess the effect of different parameterisations of two components of the modelling procedure: (i) urban-rural adjustments and (ii) estimated proportion of fevers presenting to clinics harbouring a *P. falciparum* infection. In both cases the modelling procedure was re-run under alternative configurations, producing estimates that could be compared to those from the main model run, and these results are presented in Table S3.1. In each case, estimates were made of the total number of paediatric fevers attending public health facilities likely to be accompanied by a *P. falciparum* infection (columns '*Pf+* all' in Table S3.1), and this estimate was further stratified by areas of low or moderate (*Pf*PR_2-10_ < 40%, listed in columns '*Pf*+ low' in Table S3.1) and high (*Pf*PR_2-10_ ≥40%, listed in columns '*Pf*+ high' in Table S3.1) endemicity. Results from the full model used to generate results for this study are also presented in Table S3.1 for reference ('U-R adjusted, positivity rate = median').

**Assessing the effect of urban-rural adjustment**

Protocol S1 describes the rationale and methodology by which fever prevalence and treatment seeking proportions reported at ADMIN1 level were adjusted on a pixel-by-pixel basis to reflect nationally reported urban-rural differentials whilst retaining the population-weighted mean at ADMIN1 level. To test the extent to which this adjustment affected output estimates, the model was re-run using the unadjusted ADMIN1 level fever prevalence and treatment seeking proportions directly (Table S3.1, 'No urban-rural adjustment'). The overall effect of the urban-rural adjustment was modest, with a mean national level difference in the total number of presenting *P. falciparum* positive fevers of just under 1%, although this value varied considerably between countries (Table S3.1). The largest effect was observed for Somalia where incorporating an urban-rural adjustment increased the estimate by 14%.

**Incorporating variation in the proportion of fevers presenting to clinics with confirmed malaria independent of transmission setting**

Protocol S2 describes the assembly of reported data on the proportion of paediatric fever cases presenting to the formal health sector who were confirmed positive for *P. falciparum*. Figure 3 of the main manuscript displays the observed variation in this proportion within and between three levels of transmission intensity, defined as *Pf*PR_2-10_ ≤5%, >5% to ≤40% and >40%. To test the effect of incorporating this variation on our estimates, the model was re-run using the 0.25 and 0.75 quantile of this proportion for each endemicity class rather than the median. These results are displayed in Table S3.1 under the headings ‘U-R adjusted, positivity rate = LIQR’ and ‘U-R adjusted, positivity rate = UIQR’ respectively. Using the 0.25 quantile proportion for all endemicity classes resulted in a mean national level reduction of 31%, 36% and 20% in estimates of total *P. falciparum* positive fevers attending clinics in all areas, low or moderate endemicity areas, and high endemicity areas respectively. Using the 0.75 quantile proportion led to increases of 53%,63% and 27% in the equivalent estimates.

| Country^i^ | Fevers | U-R adjusted, positivity rate = median | | |  | U-R adjusted, positivity rate = LIQR | | |  | U-R adjusted, positivity rate = UIQR | | |  | No urban-rural adjustment | | |
| --- | --- | --- | --- | --- | --- | --- | --- | --- | --- | --- | --- | --- | --- | --- | --- | --- |
|  |  | *Pf*+ all | *Pf*+ low | *Pf*+ high |  | *Pf*+ all | *Pf*+ low | *Pf*+ high |  | *Pf*+ all | *Pf*+ low | *Pf*+ high |  | *Pf*+ all | *Pf*+ low | *Pf*+ high |
|  |  |  |  |  |  |  |  |  |  |  |  |  |  |  |  |  |
| Angola | 6,176 | 3,299 | 286 | 3,013 |  | 2,595 | 195 | 2,400 |  | 4,244 | 414 | 3,830 |  | 3,285 | 287 | 2,998 |
| Benin | 1,934 | 1,116 | 52 | 1,064 |  | 884 | 36 | 847 |  | 1,423 | 71 | 1,352 |  | 1,114 | 54 | 1,060 |
| Burkina Faso | 1,499 | 884 | 1 | 883 |  | 704 | 1 | 703 |  | 1,124 | 1 | 1,123 |  | 889 | 1 | 888 |
| Burundi | 1,407 | 401 | 301 | 99 |  | 290 | 211 | 79 |  | 540 | 414 | 126 |  | 398 | 304 | 94 |
| Cameroon | 1,971 | 1,061 | 157 | 905 |  | 830 | 109 | 721 |  | 1,365 | 215 | 1,150 |  | 1,051 | 159 | 892 |
| CAR^ii^ | 933 | 541 | 21 | 521 |  | 429 | 14 | 415 |  | 690 | 28 | 662 |  | 545 | 18 | 527 |
| Chad | 1,827 | 660 | 631 | 29 |  | 459 | 436 | 23 |  | 924 | 888 | 37 |  | 669 | 643 | 27 |
| Comoros | 39 | 1 | 1 | 0 |  | 0 | 0 | 0 |  | 4 | 4 | 0 |  | 1 | 1 | 0 |
| Congo | 690 | 407 | 0 | 407 |  | 324 | 0 | 324 |  | 518 | 0 | 518 |  | 408 | 0 | 408 |
| Côte D'Ivoire | 2,132 | 1,247 | 0 | 1,247 |  | 993 | 0 | 993 |  | 1,585 | 0 | 1,585 |  | 1,261 | 0 | 1,261 |
| Djibouti | 131 | 4 | 4 | 0 |  | 1 | 1 | 0 |  | 14 | 14 | 0 |  | 4 | 4 | 0 |
| DRC^ii^ | 20,969 | 11,335 | 471 | 10,864 |  | 8,983 | 329 | 8,654 |  | 14,457 | 647 | 13,810 |  | 11,339 | 478 | 10,861 |
| Eq. Guinea | 95 | 54 | 4 | 49 |  | 42 | 3 | 39 |  | 69 | 6 | 63 |  | 54 | 4 | 49 |
| Ethiopia | 6,939 | 578 | 578 | 0 |  | 349 | 349 | 0 |  | 1,019 | 1,019 | 0 |  | 592 | 592 | 0 |
| Gabon | 208 | 117 | 14 | 102 |  | 91 | 10 | 81 |  | 150 | 20 | 130 |  | 116 | 14 | 102 |
| Gambia | 154 | 63 | 63 | 0 |  | 44 | 44 | 0 |  | 86 | 86 | 0 |  | 63 | 63 | 0 |
| Ghana | 3,847 | 2,169 | 110 | 2,058 |  | 1,717 | 77 | 1,640 |  | 2,768 | 151 | 2,616 |  | 2,178 | 110 | 2,068 |
| Guinea | 3,123 | 1,645 | 460 | 1,186 |  | 1,266 | 321 | 945 |  | 2,139 | 631 | 1,507 |  | 1,667 | 460 | 1,207 |
| Guinea Bissau | 113 | 46 | 46 | 0 |  | 32 | 32 | 0 |  | 63 | 63 | 0 |  | 45 | 45 | 0 |
| Kenya | 11,821 | 1,724 | 1,502 | 222 |  | 1,148 | 971 | 177 |  | 2,672 | 2,390 | 282 |  | 1,722 | 1,500 | 222 |
| Liberia | 1,257 | 735 | 0 | 735 |  | 585 | 0 | 585 |  | 934 | 0 | 934 |  | 737 | 0 | 737 |
| Madagascar | 3,629 | 1,623 | 775 | 847 |  | 1,216 | 541 | 675 |  | 2,145 | 1,068 | 1,077 |  | 1,624 | 777 | 847 |
| Malawi | 1,668 | 857 | 295 | 563 |  | 654 | 206 | 448 |  | 1,120 | 405 | 715 |  | 878 | 284 | 595 |
| Mali | 2,507 | 1,334 | 119 | 1,214 |  | 1,048 | 80 | 967 |  | 1,720 | 177 | 1,544 |  | 1,346 | 122 | 1,225 |
| Mauritania | 85 | 12 | 11 | 1 |  | 7 | 7 | 1 |  | 21 | 20 | 1 |  | 12 | 11 | 1 |
| Mozambique | 10,360 | 5,202 | 1,056 | 4,145 |  | 4,031 | 729 | 3,302 |  | 6,758 | 1,488 | 5,269 |  | 5,237 | 1,041 | 4,196 |
| Namibia | 423 | 51 | 51 | 0 |  | 30 | 30 | 0 |  | 92 | 92 | 0 |  | 51 | 51 | 0 |
| Niger | 4,480 | 1,923 | 1,321 | 602 |  | 1,397 | 918 | 480 |  | 2,602 | 1,836 | 766 |  | 1,924 | 1,324 | 601 |
| Nigeria | 35,781 | 20,345 | 1,540 | 18,805 |  | 16,056 | 1,076 | 14,980 |  | 26,019 | 2,115 | 23,905 |  | 20,559 | 1,516 | 19,043 |
| Rwanda | 2,170 | 593 | 579 | 14 |  | 416 | 405 | 11 |  | 813 | 796 | 18 |  | 593 | 579 | 14 |
| Senegal | 4,165 | 1,269 | 1,269 | 0 |  | 883 | 883 | 0 |  | 1,759 | 1,759 | 0 |  | 1,272 | 1,272 | 0 |
| Sierra Leone | 2,164 | 1,233 | 0 | 1,233 |  | 982 | 0 | 982 |  | 1,568 | 0 | 1,568 |  | 1,235 | 0 | 1,235 |
| Somalia | 87 | 21 | 19 | 1 |  | 14 | 13 | 1 |  | 31 | 29 | 2 |  | 24 | 22 | 2 |
| ST & P^ii^ | 15 | 6 | 6 | 0 |  | 4 | 4 | 0 |  | 8 | 8 | 0 |  | 6 | 6 | 0 |
| Sudan | 12,563 | 1,786 | 1,742 | 45 |  | 1,090 | 1,055 | 36 |  | 3,120 | 3,064 | 57 |  | 1,785 | 1,742 | 43 |
| Swaziland | 371 | 26 | 26 | 0 |  | 16 | 16 | 0 |  | 44 | 44 | 0 |  | 26 | 26 | 0 |
| Tanzania | 15,140 | 5,952 | 3,496 | 2,456 |  | 4,365 | 2,408 | 1,957 |  | 8,068 | 4,946 | 3,122 |  | 5,923 | 3,464 | 2,459 |
| Togo | 525 | 310 | 0 | 310 |  | 247 | 0 | 247 |  | 394 | 0 | 394 |  | 310 | 0 | 310 |
| Uganda | 13,002 | 5,899 | 3,156 | 2,743 |  | 4,390 | 2,205 | 2,185 |  | 7,821 | 4,335 | 3,486 |  | 5,885 | 3,157 | 2,728 |
| Zambia | 5,049 | 1,746 | 1,612 | 133 |  | 1,215 | 1,108 | 106 |  | 2,460 | 2,291 | 170 |  | 1,757 | 1,622 | 135 |
| Zimbabwe | 985 | 34 | 34 | 0 |  | 12 | 12 | 0 |  | 93 | 93 | 0 |  | 33 | 33 | 0 |
| **Total** | **182,433** | **78,306** | **21,810** | **56,496** |  | **59,841** | **14,836** | **45,006** |  | **103,446** | **31,629** | **71,817** |  | **78,619** | **21,785** | **56,834** |
| i. Four countries excluded due to unavailable data: Eritrea, South Africa, Botswana and Cape Verde. ii. CAR: Central African Republic; DRC: Democratic Republic of Congo; ST & P: São Tomé and Principe. | | | | | | | | | | | | | | | | |

**Table S3.1. Total and *P. falciparum* positive paediatric fevers attending public health facilities: estimate under four different model configurations.** All values in '000s. See text for further explanation.
