# Supplementary material for: Estimating the Number of Paediatric Fevers Associated with Malaria Infection Presenting to Africa's Public Health Sector in 2007
Source: PLoS Med. 2010 Jul 6;7(7):e1000301. doi: 10.1371/journal.pmed.1000301 (PMC2897768; doi:10.1371/journal.pmed.1000301)
Supplement: Table S1 — Data assembled to define period prevalence of fever among children 0–4 y at subnational levels from 42 African national sample surveys. (0.08 MB DOC) [file pmed.1000301.s004.doc]

| **Country** | **ACT policy** | **Source/ admin units** | **Survey Date** | **Children seen, with fever (%)** | **Fevers seen at clinic (%)** |
| --- | --- | --- | --- | --- | --- |
|  |  |  |  |  |  |
| Angola | AL | MIS (4) | Nov-Apr 2006-07 | 2385, 534 (24.2%) | 233 (43.6%) |
| Benin | AL | DHS (12) | Jul-Nov 2006 | 14,682, 4204 (28.6%) | 1019 (26.0%) |
| Botswana | AL | - | - | - | - |
| Burkina Faso | AL | MICS (13) | Apr-Jun 2006 | 5677, 1051 (18.5%) | 134 (12.7%) |
| Burundi | AQAS | MICS (5) | Sep-Dec 2005 | 6934, 1923 (27.7%) | 361 (18.8%) |
| Cameroon | AQAS | MICS (10) | May-Jun 2006 | 6495, 1154 (17.8%) | 243 (21.1%) |
| Cape Verde | AL | - | - | - | - |
| CAR | AL | DHS (6) | Sep-Mar 1994-95 | 2561, 866 (33.8%) | 245 (28.3%) |
| Chad | AQAS | DHS (8) | Jul-Dec 2004 | 4926, 1547 (31.4%) | 240 (15.5%) |
| Comoros^1^ | AL | MICS (3) | Oct-Dec 2000 | 4870, 491 (10.1%) | 231 (47.0%) |
| Congo | AQAS | DHS (4) | Jul-Nov 2005 | 4435, 1002 (22.6%) | 431 (43.0%) |
| Côte D’Ivoire | AQAS | MICS (11) | Aug-Oct 2006 | 8604, 2211 (25.7%) | 311 (14.1%) |
| Djibouti | SPAS | MIS (5) | Dec-Feb 2008-09 | 3022, 598 (19.8%) | 244 (40.9%) |
| DRC | AQAS | DHS (11) | May-Aug 2007 | 7987, 2556 (32.0%) | 670 (26.2%) |
| Eq. Guinea^1^ | AQAS | MICS (7) | Jul-Nov 2000 | 2457, 739 (30.1%) | 140 (19.0%) |
| Eritrea | AQAS | - | - | - | - |
| Ethiopia | AL | DHS (11) | Apr-Aug 2005 | 9002, 1587 (17.6%) | 231 (14.6%) |
| Gabon | AQAS | DHS (5) | Jul-Jan 2000-01 | 4100, 1163 (28.4%) | 275 (23.6%) |
| Gambia | AL | MICS (7) | Mar-Dec 2005-06 | 6641, 538 (8.1%) | 219 (40.7%) |
| Ghana | AQAS | MICS (10) | Aug-Oct 2006 | 3545, 796 (22.5%) | 230 (28.9%) |
| Guinea | AQAS | DHS (8) | Feb-Jun 2005 | 5641, 1791 (31.7%) | 524 (29.3%) |
| Guinea Bissau | AL | MICS (4) | May-Jun 2006 | 6570, 787 (12.0%) | 181 (23.0%) |
| Kenya^2^ | AL | MIS (8) | Jun-Jul 2007 | 6373, 1731 (27.1%) | 582 (33.6%) |
| Liberia | AQAS | DHS (15) | Dec-Apr 2006-07 | 5305, 1673 (31.5%) | 663 (21.0%) |
| Madagascar | AQAS | DHS (6) | Nov-Mar 2003-04 | 5088, 1040 (20.4%) | 270 (26.0%) |
| Malawi^3^ | AL | MICS (3) | Jul-Nov 2006 | 23238, 8057 (34.7%) | 773 (9.6%) |
| Mali | AL | DHS (9) | Apr-Sep 2006 | 12437, 2094 (16.8%) | 600 (28.7%) |
| Mauritania^1^ | AQAS | MICS (13) | May-Sep 2007 | 8981, 1445 (16.1%) | 162 (11.2%) |
| Mozambique | AL | DHS (11) | Aug-Dec 2003 | 9129, 2322 (25.4%) | 1203 (51.8%) |
| Namibia | AL | DHS (13) | Oct-Mar 2006-07 | 4858, 773 (15.9%) | 394 (51.0%) |
| Niger | AL | DHS (8) | Jan-Jun 2006 | 8209, 2170 (26.4%) | 711 (32.8%) |
| Nigeria | AL | DHS (6) | Mar-Aug 2003 | 5186, 1603 (30.9%) | 386 (24.1%) |
| Rwanda | AL | DHS (12) | Feb-Jul 2005 | 7752, 2001 (25.8%) | 476 (23.8%) |
| ST & P^1^ | AQAS | MICS (2) | Jan-Mar 2000 | 2209, 103 (4.7%) | 40 (38.8%) |
| Senegal^4^ | AQAS | MIS (10) | Dec-Jan 2008-9 | 13316, 4123 (31.0%) | 1578 (38.2%) |
| Sierra Leone^1^ | AQAS | MICS (4) | Oct-Nov 2005 | 5904, 1829 (31.0%) | 701 (38.3%) |
| Somalia | SPAS | MICS (3) | Aug-Sep 2006 | 6373, 1288 (20.2%) | 14 (1.1%) |
| South Africa | AL/ AQAS^5^ | - | - | - | - |
| Sudan (Nth)^1,6^ | SPAS | SHHS (16) | Oct-Nov 2006 | 12238, 1423 (11.6%) | 982 (69.1%) |
| Sudan (Sth)^1^ | AL | SHHS (10) | Oct-Nov 2005 | 7287, 3316 (45.5%) | 1660 (50.0%) |
| Swaziland | AL | DHS (4) | Jul-Feb 2006-07 | 2537, 712 (28.1%) | 312 (43.8%) |
| Tanzania (M)^7^ | AL | AIS-MIS (20) | Oct-Feb 2007-08 | 5141, 971 (18.9%) | 495 (51.0%) |
| Tanzania (Z)^7^ | AQAS | AIS-MIS (2) | Oct-Feb 2007-08 | 1890, 229 (12.1%) | 155 (67.7%) |
| Togo^1,8^ | AL | MICS (5) | May-Jun 2006 | 4154, 766 (18.4%) | 119 (15.5%) |
| Uganda | AL | DHS (9) | May-Oct 2006 | 7593, 3091 (40.7%) | 924 (29.9%) |
| Zambia | AL | DHS (9) | Apr-Oct 2007 | 5844, 1034 (17.7%) | 602 (58.2%) |
| Zimbabwe | AL | DHS (10) | Aug-Mar 2005-06 | 4875, 391 (8.0%) | 122 (31.2%) |
|  |  |  |  |  |  |
| 1. Data on the treatment seeking behavior/sources of treatment for ARI/Pneumonia used as a proxy for sources of treatment for fever; 2. Nairobi Province was not included in the 2007 MIS for Kenya and therefore data used from the 2003 DHS as a replacement; 3. Treatment sources not available in the reports from this survey so used fever treatment sources derived for the same ADMIN1 units during the DHS 2000 in Malawi; 4.Treatment sources not available for the MIS 2008-9 in the report so used the treatment sources for documented fevers in the same ADMIN1 units derived from the MIS in November-December 2006;  5. AL policy operational in Kwa-Zulu Natal Province & ASAQ policy in Mpulanganga Province; 6. In Sudan (North) no data were available for West Kordofan and there were no replacement data for this administrative unit; 7. Separate data provided for Tanzania mainland (M) and Zanzibar (Z); 8. During the Togo MICS in 2006 two provinces (Plateaux and Savanes) lacked reports on treatment seeking for ARI so we have averaged the proportion accessing a public health facility from the three ADMIN1 units where data were provided. | | | | | |

**Table S1. Data assembled to define period prevalence of fever among children 0-4 years at sub-national levels from national sample surveys in 42/47 African countries.** Information on currently recommended first line drug policies derived from WHO Global Malaria Programme (11 May 2009 & Swazliland NMCP who are implementing AL policy change late 2009): Artemether Lumefanthrine (AL); Amodiaquine-Artesunate (AQAS); suplhadoxine-pyrimethamine-Artesunate (SPAS); Central African Republic (CAR); Democratic Republic of Congo (DRC); São Tomé and Principe (ST & P).
